# Clinical Practice Guideline for the Management of Non-chemotherapeutic Extravasation: A Multidisciplinary Delphi-driven Consensus Study

**DOI:** 10.5812/ijpr-171391

**Published:** 2026-09-09

**Authors:** Sima Mollaeian, Behzad Azimi, Ghader Mohammadnezhad, Seyydeh Fatemeh Mousavi, Behnam Molavi, Pariya Kalani, Hadi Esmaily

**Affiliations:** 1 Department of Clinical Pharmacy, School of Pharmacy, Shahid Beheshti University of Medical Sciences 1996835113 Tehran Iran; 2 Department of Vascular and Endovascular Surgery, Imam Hossein Educational Hospital, Shahid Beheshti University of Medical Sciences 1617763141 Tehran Iran; 3 Clinical Research Development Center, Imam Hossein Educational Hospital, Shahid Beheshti University of Medical Sciences 1617763141 Tehran Iran; 4 Neonatal Intensive Care Unit, Imam Hossein Educational Hospital, Shahid Beheshti University of Medical Sciences 1617763141 Tehran Iran; 5 Department of Surgery, Shariati Hospital, Tehran University of Medical Sciences 1411713135 Tehran Iran

**Keywords:** Clinical Practice Guideline, Delphi Method, Drug Extravasation, Non-chemotherapeutic Agents, Patient Safety

## Abstract

**Background:**

Extravasation, the unintended leakage of an intravenous drug into surrounding tissue, is a serious complication of parenteral therapy that can cause skin necrosis, functional impairment, or limb loss. Most existing guidelines focus on chemotherapeutic agents; however, serious injury also occurs with non-chemotherapeutic drugs, and comprehensive guidance for these agents is lacking.

**Objectives:**

To develop a structured, evidence-based guideline to support the prevention, early recognition, and management of nonchemotherapy drug extravasation.

**Methods:**

The initial draft was informed by a systematic review of studies published between 2000 and September 2024, conducted in accordance with the Preferred Reporting Items for Systematic Reviews and Meta-Analyses guidelines, and by an analysis of existing protocols. The subsequent three-round Delphi consensus process was conducted in accordance with the Conducting and REporting of DElphi Studies guidelines.

**Results:**

The panel comprised six pharmacists, three vascular surgeons, and four expert nurses. Consensus (≥ 70%) was reached on 20 final items. The guideline addresses the mechanisms of tissue injury, major complications, and preventive strategies. It also addresses previously underexplored areas, including drug risk stratification, early detection technologies, appropriate catheter selection for prevention, and emerging therapeutic approaches that require further evaluation. This guideline is intended to equip frontline clinicians (nurses, pharmacists, and surgeons) to support patient safety and standardize care for vulnerable populations receiving intravenous therapy; its effects on injury incidence and severity should be confirmed in future implementation studies.

**Conclusions:**

This structured, consensus-based guideline incorporates novel domains and updates prior protocols. It may help standardize the recognition and management of non-chemotherapeutic drug extravasation; however, its clinical impact should be evaluated in future implementation studies.

## 1. Background

Extravasation, the unintended leakage of a drug into surrounding tissue during intravenous administration, is a significant complication that can cause skin necrosis, limb dysfunction, compartment syndrome, or, in severe cases, amputation. Up to 6% of intravenous injections may result in some degree of extravasation (1), highlighting the need for prompt prevention and early intervention. Timely management often prevents major injury, whereas delays in diagnosis or treatment can cause irreversible damage, such as deep tissue necrosis or limb amputation (2). Extravasation also prolongs hospital stays, increases healthcare costs, and undermines patient trust (3-5).

This issue affects nurses, clinical pharmacists, dermatologists, vascular surgeons, and general practitioners, all of whom require clear guidance. Injury severity depends on the drug’s physicochemical properties (pH, osmolality, and cellular toxicity), the injection site, infusion rate, and patient-specific characteristics (6). Vulnerable groups, such as intensive care patients, neonates, older adults, and patients with malignancy, face higher risks because of fragile vessels and tissues (1). Extravasation occurs through direct needle perforation or endothelial damage (7). Common sites include the dorsum of the hand, forearm, and foot, where thin skin overlies commonly used venous access sites (8).

Early signs, such as tingling, erythema, pain, or blistering, may appear mild; however, deeper damage is often not immediately visible, leading to an underestimation of severity (7, 9). Despite its clinical significance, most guidelines focus on chemotherapeutic or cytotoxic agents, although non-chemotherapeutic drugs can also cause serious tissue injury. In this guideline, the terms “non-chemotherapeutic,” “noncytotoxic,” and “non-cytotoxic” are used interchangeably to describe agents other than antineoplastic or chemotherapeutic drugs.

## 2. Objectives

To provide an updated, expert-informed protocol for non-chemotherapeutic drug extravasation that incorporates recent evidence on preventive strategies, including appropriate catheter selection (10); diagnostic tools, including bioimpedance, thermal, and optical detectors (11); and emerging investigational treatments, including the amniotic membrane, vacuum-assisted closure (VAC), hyperbaric oxygen therapy (HBO), and active Leptospermum honey (6, 12-15).

## 3. Methods

This study was designed as a systematic-review-informed, Delphi-consensus, guideline-development study comprising three linked components governed by distinct reporting standards: an evidence-review phase reported in accordance with the Preferred Reporting Items for Systematic Reviews and Meta-Analyses (PRISMA) 2020 Statement; a Delphi consensus process reported in accordance with the Conducting and REporting of DElphi Studies (CREDES) guideline; and development of the final clinical practice guideline reported according to the Reporting Items for Practice Guidelines in Healthcare (RIGHT) Statement.

### 3.1. Systematic Review Protocol for Drafting the Initial Guideline

Previous guidelines were assessed to identify gaps, which informed an initial topic list. Keywords and search strategies were then developed. Two authors independently searched PubMed (MEDLINE), Scopus, and Web of Science (Clarivate) for drug extravasation studies published in English between 2000 and September 2024. The search strategies used Boolean operators and truncation (detailed in Supplementary File 1). The search was conducted to inform the development of the initial guideline draft that served as the starting point for the Delphi consensus process, rather than as a continuously updated systematic evidence review. After automated duplicate removal using EndNote, followed by manual verification, two authors independently screened titles and abstracts and subsequently assessed potentially eligible full-text articles against the predefined eligibility criteria. Studies were excluded if they involved non-human subjects, lacked full-text availability, were unrelated to intravenous extravasation, or focused on physiological cell migration. Disagreements between the two independent reviewers were resolved through discussion until consensus was reached. The systematic review was conducted and reported in accordance with the PRISMA 2020 Statement (16), which served as the reporting guideline for this evidence review. Relevant information from the included studies was independently extracted by two authors and organized according to the predefined guideline topics. For each guideline domain, including risk factors, clinical manifestations, prevention, diagnosis, and management, evidence from studies addressing the respective topic was used to draft the preliminary guideline statements. A formal risk-of-bias assessment was not performed.

### 3.2. Certainty of Evidence

Evidence certainty was assessed qualitatively. Because of the heterogeneous evidence base (guidelines, case reports, expert opinion, and systematic reviews), formal application of the GRADE framework was considered inappropriate. The research team therefore used a qualitative evidence-grading approach rather than a GRADE assessment. Accordingly, systematic reviews were considered to provide high-to-moderate-certainty evidence (Grade B), whereas clinical guidelines and narrative reviews were considered to provide moderate-to-low-certainty evidence (Grade C). Individual case reports were considered low-certainty evidence (Grade D). A formal risk-of-bias assessment or quality-appraisal tool was not applied, which is acknowledged as a limitation of the evidence base underlying the guideline recommendations.

### 3.3. Recommendation Strength

Recommendation strength was pragmatically defined as strong (> 3 supporting sources), moderate (2 supporting sources), or weak (1 supporting source). This approach was adopted because the objective of the literature review was to inform Delphi-based guideline development rather than to perform a formal evidence synthesis for individual clinical questions. Recommendation strength was not intended to represent GRADE recommendation strength; rather, it was used as a pragmatic indicator reflecting the amount of available supporting evidence during Delphi-based guideline development.

The available evidence comprised diverse publication types, including systematic reviews, clinical guidelines, expert consensus documents, narrative reviews, and case reports. Accordingly, GRADE domains (risk of bias, inconsistency, indirectness, imprecision, and publication bias) were not formally evaluated for each recommendation. Evidence-certainty grades (B-D) were assigned at the level of individual recommendations rather than at the level of the 20 guideline sections. Because each section contains multiple recommendations supported by different types and levels of evidence, assigning a single evidence-certainty grade to an entire section would be misleading. Therefore, Table 1 reports only the Delphi consensus achieved for each guideline section, whereas certainty-of-evidence and recommendation-strength grades for individual recommendations are reported in Appendix A, Table 13 in the Supplementary File (Conservative Treatment Protocols by Drug Classification).

**Table 1.** Expert Consensus (%) for Items Across Delphi Rounds ^a^

Guideline Item	Round 1 Agreement	Round 2 Agreement	Round 3 Agreement	Final Status	Modifications in Delphi
**Introduction**	76.9 (10/13)	81.8 (9/11)	81.8 (9/11)	Accepted	Unanimously approved in Round 1.
**Key definitions of extravasation**	76.9 (10/13)	81.8 (9/11)	81.8 (9/11)	Accepted	Clarified with added definitions.
**Clinical signs**	100 (13/13)	100 (11/11)	100 (11/11)	Accepted	Fully endorsed in Round 1.
**Mechanisms of tissue injury**	76.9 (10/13)	81.8 (9/11)	81.8 (9/11)	Accepted	Suggested a table grouping drugs by injury mechanism.
**Complications**	84.6 (11/13)	81.8 (9/11)	81.8 (9/11)	Accepted	Enhanced with new items for clarity.
**Risk factors**	100 (13/13)	100 (11/11)	100 (11/11)	Accepted	Gained full approval in Round 1.
**Preventive strategies**	100 (13/13)	100 (11/11)	100 (11/11)	Accepted	Approved in Round 1 with minor wording tweaks.
**Diagnostic approaches**	100 (13/13)	100 (11/11)	100 (11/11)	Accepted	Unanimously supported in Round 1.
**Differential diagnoses**	76.9 (10/13)	81.8 (9/11)	81.8 (9/11)	Accepted	Deemed non-critical by some, but met the threshold.
**Components of an extravasation kit**	100 (13/13)	100 (11/11)	100 (11/11)	Accepted	Fully agreed upon in Round 1.
**Immediate nursing actions**	100 (13/13)	100 (11/11)	100 (11/11)	Accepted	Unanimously accepted in Round 1.
**Pharmacologic treatment**	100 (13/13)	100 (11/11)	100 (11/11)	Accepted	Added a table for antidote access; fully endorsed in Round 1.
**Non-pharmacologic treatment**	100 (13/13)	100 (11/11)	100 (11/11)	Accepted	Reworded for scientific precision, achieving full consensus.
**Recommended interventions for each drug**	100 (13/13)	100 (11/11)	100 (11/11)	Accepted	Fully supported in Round 1.
**Documentation**	84.6 (11/13)	90.9 (10/11)	90.9 (10/11)	Accepted	Seen as less urgent by some, but met consensus.
**Patient follow-up**	92.3 (12/13)	100 (11/11)	100 (11/11)	Accepted	Revised for scientific clarity, increasing agreement.
**Detector**	Added after Round 1	90.9 (10/11)	100 (11/11)	Accepted	Wording refined to boost agreement.
**Catheter selection**	Added after Round 1	72.7 (8/11)	72.7 (8/11)	Accepted	Some viewed as outside scope; text revised.
**Proposed treatment strategies**	Added after Round 1	81.8 (9/11)	100 (11/11)	Accepted	Included based on recent studies and experience.
**A risk-based classification table**	Added after Round 1	100 (11/11)	100 (11/11)	Accepted	Reworded for precision, reaching greater consensus.

^a^ Values are expressed as percentages (ratios). Consensus was predefined as ≥ 70% agreement. Respondents per round: Round 1, n = 13; Round 2, n = 11; Round 3, n = 11. Agreement percentages, including for the Recommended Interventions for Each Drug section, reflect consensus on the corresponding guideline section as a whole and should not be interpreted as separate item-level consensus for each individual drug-specific recommendation.

All recommendations were reviewed and validated by the expert panel and practicing nurses to ensure clinical applicability. The study selection process is summarized in Figure 1.

**Figure 1.**
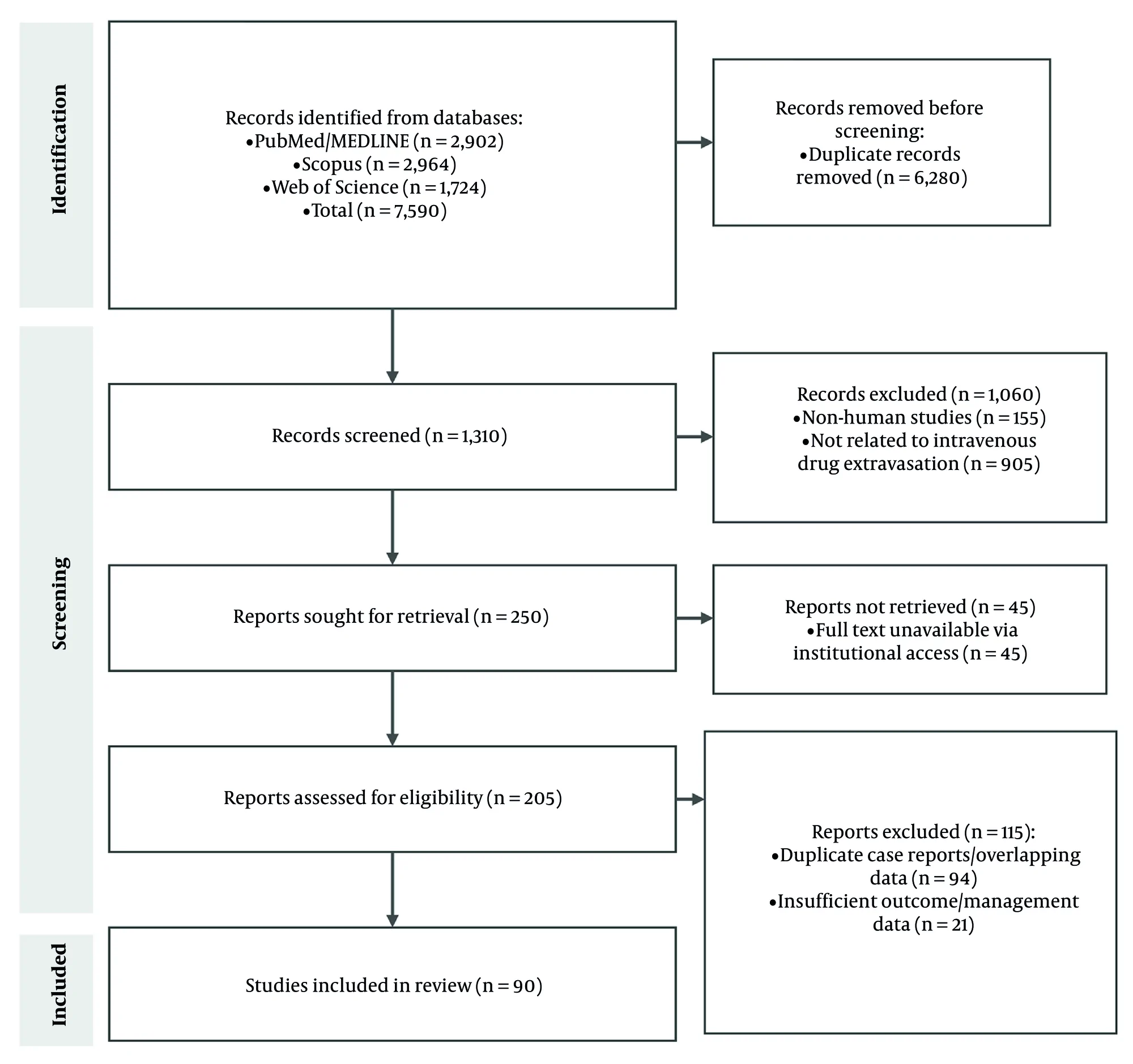
PRISMA 2020 flow diagram of the study identification and selection process.

### 3.4. Study Design

This study used the Delphi method, a structured and practical approach to building expert consensus on non-chemotherapeutic drug extravasation (17, 18). The Delphi technique, known for its reproducibility and iterative process, enables the collection of diverse expert opinions through repeated feedback cycles (17, 18). The three-round process followed the CREDES checklist. No patients were involved.

### 3.5. Expert Panel

Experts were recruited using purposive sampling based on their professional discipline, years of clinical experience, and direct involvement in the prevention and management of extravasation. Eligible panel members had professional experience in extravasation management, teaching expertise, membership in hospital committees related to drug administration, formal training in this field, and demonstrated professional competence. Potential panel members were identified by the research team in consultation with senior clinicians experienced in extravasation management, using predefined eligibility criteria to ensure multidisciplinary representation and relevant clinical expertise. All Delphi panel members completed a conflict-of-interest declaration, and no relevant conflicts of interest were declared. Panel members evaluated the accuracy of the information and proposed interventions (19-21). Based on Delphi methodology, a panel of 10 to 15 members is considered sufficient (20-22). Because increasing the panel size beyond this range has minimal impact on statistical power or validity, 13 experts were invited, and all agreed to participate (19). The panel comprised 3 vascular surgeons, 6 clinical pharmacy specialists, and 4 nurses. Because two panel members were unavailable after Round 1, Rounds 2 and 3 proceeded with 11 panel members (Table 2). The remaining panel retained representation from all three professional disciplines (vascular surgery, clinical pharmacy, and nursing) and remained within the recommended Delphi panel size; therefore, attrition was not considered to have materially affected the consensus process. All participants provided informed consent.

**Table 2.** Expert Panel Composition and Delphi Round Participation

Professions	Backgrounds	Attendance in Delphi Rounds
**Physician**	3 vascular surgeons: 2 from Imam Hossein Hospital and 1 from Shariati Hospital, Tehran. Each had more than 10 years of experience in the prevention and management of extravasation injuries.	2 attended all three rounds; 1 attended only Round 1.
**Pharmacist**	6 clinical pharmacy specialists: Including 3 university faculty members. Four belonged to the Clinical Pharmacy Care Committee at Imam Hossein Hospital.	All participated in all three rounds.
**Nurse**	4 nurses: All were employed at Imam Hossein Hospital and had at least five years of experience in the prevention and management of extravasation injuries.	3 attended all three rounds; 1 attended only Round 1.

### 3.6. Delphi Approach

The authors developed a draft framework based on prior studies and refined it through expert feedback. Participant responses remained anonymous throughout all Delphi rounds. After each round, the research team summarized the overall level of agreement together with anonymized participant comments. The guideline draft was revised accordingly; newly proposed topics were incorporated into the subsequent Delphi round for formal evaluation, whereas editorial, wording, and explanatory revisions that did not alter the content of the recommendations were incorporated into the draft and communicated to all panel members. The updated version, accompanied by aggregated agreement percentages and anonymized feedback, was then circulated before the subsequent round. This controlled feedback process enabled participants to review the aggregated agreement results together with the revisions made in response to panel feedback while preserving participant anonymity and minimizing the influence of dominant individuals on the consensus process.

Round 1 used a paper questionnaire with dichotomous (Yes/No) responses for each item. Agreement for each item was calculated as the percentage of panelists selecting Yes. Participants could also suggest new topics through free-text comments. Round 2 assessed scientific content, structure, clarity, applicability, and coherence; each domain was rated using the same dichotomous (Yes/No) format, with a dedicated field for suggested revisions. Items with ≥ 70% agreement were retained (23). Round 3 confirmed the revised guideline following incorporation of the agreed changes, and all items achieved the predefined consensus threshold. Each round used a tailored questionnaire (Supplementary File 2).

### 3.7. Consensus Definition

Consensus was defined a priori as ≥ 70% agreement, a threshold consistent with commonly applied conventions in Delphi-based guideline development (23-25). Items below this threshold would be reassessed. Although some items achieved agreement levels close to the predefined threshold, all exceeded the 70% criterion and were therefore retained. Panel comments were used to refine wording and improve clarity where appropriate. A process flowchart is provided in Supplementary File 3.

### 3.8. External Validation

After finalizing the draft, three independent specialists (a clinical pharmacy specialist, a nursing faculty member, and a vascular surgeon) who were not involved in the Delphi process independently evaluated the guideline using a structured validation questionnaire assessing five predefined domains: clarity, coherence, practical applicability, completeness, and structure/format (Supplementary File 4). Each domain was assessed using dichotomous Yes/No responses, with an additional free-text section for comments and suggested revisions. Quantitative agreement for each evaluation domain was calculated as the proportion of reviewers selecting Yes, and all qualitative comments were reviewed and incorporated. This external evaluation was designed as a complementary quality check of the finalized guideline following the Delphi-based consensus process, which constituted the primary methodology for guideline development.

Guideline development adhered to the RIGHT checklist.

### 3.9. Ethical Approval

The study received approval from the Research Ethics Committee of Shahid Beheshti University of Medical Sciences, Tehran, under the ethical code IR.SBMU.PHARMACY.REC.1403.153.

## 4. Results

The Delphi process spanned three rounds, with 13 panel members participating in the first round and 11 in the second and third rounds. The response rate was 100% (13/13) in the first round and 84.6% (11/13) in the subsequent rounds.

### 4.1. Round 1

The panel reviewed 16 initial items, derived from a systematic review of international literature and established protocols, along with an introductory section, using a structured questionnaire. All materials were reviewed and approved by the author team before distribution to ensure clarity and minimize potential bias. All items surpassed the 70% consensus threshold and were retained. The panel proposed four additional topics for inclusion:

- A clinical pharmacist suggested adding information on early detection technologies, such as pressure, thermal, ultrasound, and impedance-based sensors, to identify tissue damage before clinical symptoms appear.

- A vascular surgeon recommended including criteria for appropriate catheter selection as a preventive measure; this item was retained after reaching 72.7% agreement, although some panelists considered catheter selection to be outside the guideline’s core scope.

- A pediatric nurse with extensive extravasation experience proposed novel, experience-based therapies, drawn from case reports and clinical recommendations, to introduce emerging conservative interventions.

- A clinical pharmacist suggested adding a risk-based classification table for medications to increase awareness and vigilance among healthcare staff, particularly nurses.

### 4.2. Round 2

All four proposed additions achieved more than 70% agreement and were retained. The Round 1 questionnaire was redistributed alongside the Round 2 questionnaire, and some items achieved higher consensus after textual revisions. Key editorial changes included:

- Adding a cautionary note on hyaluronidase use in pediatric patients and those with fragile skin, based on panel reports of increased erythema, swelling, and discomfort.

- Expanding the definition and diagnostic criteria for compartment syndrome because of its severity as a complication.

- Removing an in-depth discussion of peripherally inserted central catheters (PICCs) from the catheter-selection section because it was deemed beyond the protocol’s scope.

- Acknowledging national-level challenges in accessing certain drugs, technologies, or treatments.

The revised draft was then circulated for a third-round review.

### 4.3. Round 3

All changes were approved, with no further modifications. All items and their explanations were accepted, concluding the Delphi process. Some panel members noted a lack of clinical urgency for certain items; however, because all met the 70% consensus threshold, they were included (Table 1).

### 4.4. External Validation

All three external reviewers rated all five evaluation domains (clarity, coherence, practical applicability, completeness, and structure/format) as acceptable (100% agreement). Qualitative feedback focused on presentation rather than content and included suggestions to reorder several section titles, clarify selected terminology, and add illustrative figures to improve readability and practical implementation. These suggestions were incorporated into the final version of the guideline.

### 4.5. Final Output

During guideline development, equity, feasibility, and acceptability were considered to ensure that the recommendations were practical, fair, and acceptable for clinical use. The finalized guideline, revised for linguistic clarity, is presented in full as a systematic-review-informed, expert-consensus guidance document for clinical use (Appendix A in the Supplementary File). An overview of the guideline structure and the content of its 20 sections is presented in Table 3.

**Table 3.** Overview of the Structure and Clinical Content of the Finalized Extravasation Management Guideline ^a^

No.	Sections	Clinical Purposes
**1**	Introduction	States the guideline's scope, rationale, and intended educational use
**2**	Definitions	Standardizes key terms (extravasation, infiltration, erythema, phlebitis, conservative management, impedance sensing, swelling measurement, drug and catheter classification)
**3**	Signs and Symptoms at Different Levels of Injury	Describes clinical presentation by severity grade to support early recognition
**4**	Mechanisms of Tissue Injury	Explains the pathophysiology of tissue damage from extravasated agents
**5**	Consequences of Extravasation	Summarizes potential complications, from mild irritation to severe necrosis
**6**	Classification of Drugs Based on Risk of Tissue Injury	Stratifies agents by vesicant/irritant potential to guide vigilance
**7**	Risk Factors for Extravasation	Identifies patient-, drug-, and device-related risk factors
**8**	Prevention	Outlines key strategies for reducing extravasation incidence
**9**	Selection of Appropriate Catheter	Gives catheter-selection criteria across patient populations (general, CKD, critically ill, repeated phlebotomy, special populations)
**10**	Diagnosis	Describes the clinical approach to confirming extravasation
**11**	Detector	Reviews early-detection technologies (impedance, temperature, radiofrequency, optical, ultrasound, pressure sensors)
**12**	Differential Diagnosis	Distinguishes extravasation from mimicking conditions (venous flare, vessel irritation, venous shock, phlebitis, hypersensitivity)
**13**	Extravasation Kit	Specifies essential components of a ready-to-use response kit
**14**	Initial Therapeutic Management	Details the immediate nursing/clinical response checklist
**15**	Pharmacologic Management	Covers antidote selection and administration (hyaluronidase, sodium thiosulfate, DMSO, dexrazoxane, phentolamine, nitroglycerin, terbutaline, and antidotes of uncertain efficacy)
**16**	Non-Pharmacological Management	Covers physical/procedural interventions (warm/cold compress, manual massage, saline flush-out, liposuction, debridement/skin grafting)
**17**	Recommended Management Strategies for Each Drug	Presents the drug-specific matrix linking each agent to compress type, antidote, and evidence grade (Table 13 in the Supplementary File)
**18**	Follow-Up	Specifies post-event monitoring and follow-up care
**19**	Documentation and Patient Records	Defines required documentation practices for extravasation incidents
**20**	Investigational Treatments	Reviews emerging/investigational therapies (VAC, HBO, amniotic membrane, active Leptospermum honey) with an evidence caveat

^a^ Abbreviations: CKD, chronic kidney disease; DMSO, dimethyl sulfoxide; HBO, hyperbaric oxygen therapy; VAC, vacuum-assisted closure.

Key clinical components include risk factors and associated preventive strategies, identification of extravasation onset, a tiered classification system for assessing extravasation severity, and a quick-reference manual to support prompt management of extravasation. All recommendations presented in this algorithm are classified as Grade C (moderate-certainty evidence) (Figure 2) (1, 26).

**Figure 2.**
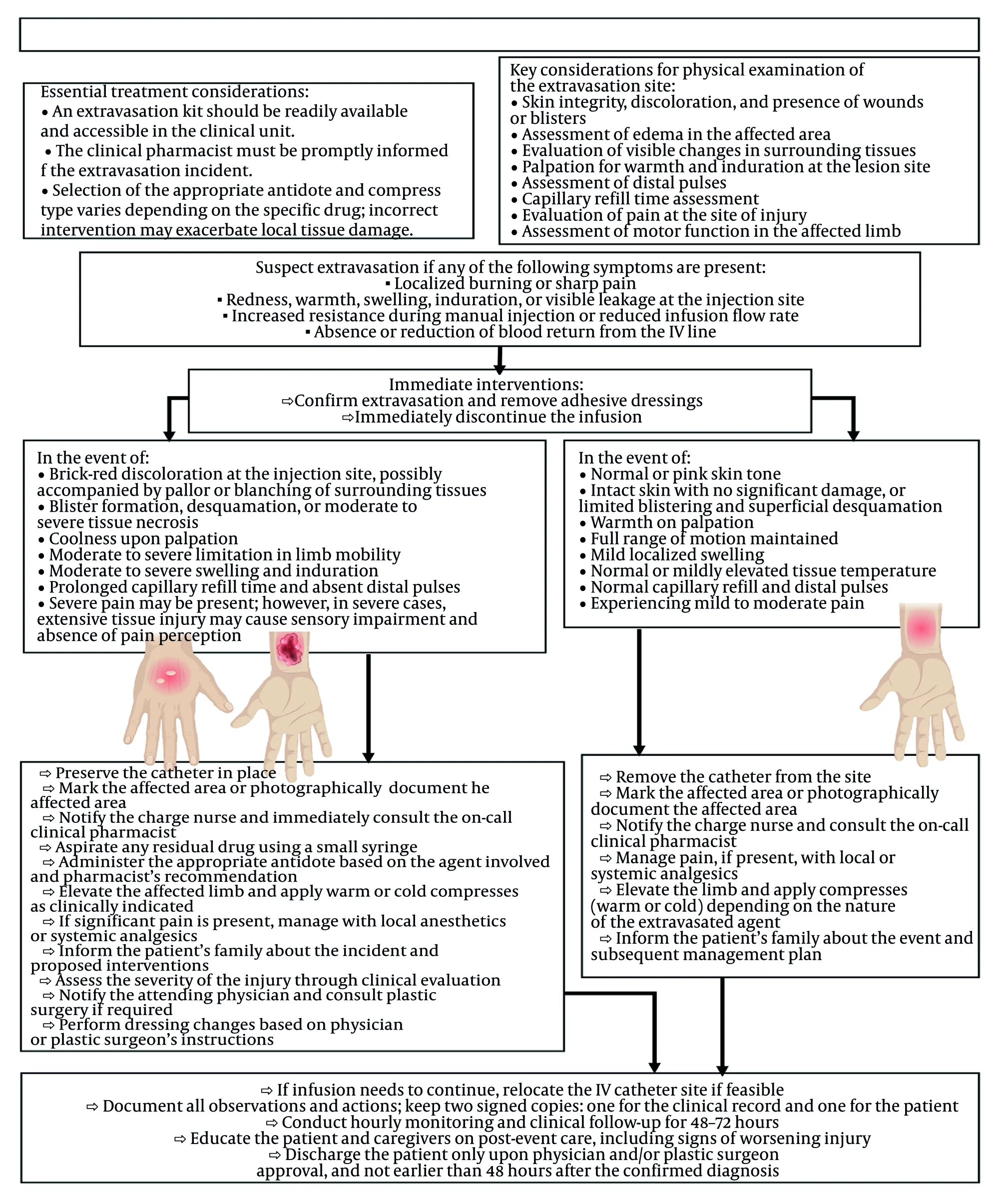
Stepwise management algorithm for the management of non-chemotherapeutic extravasation. Image provided by Servier Medical Art (https://smart.servier.com/), licensed under CC BY 4.0 (https://creativecommons.org/licenses/by/4.0/).

The guideline also includes a drug-risk/action matrix that categorizes medications according to their risk of tissue injury and outlines first-line management strategies; pharmacological and non-pharmacological treatment recommendations, including compress application, saline flush-out, and specific antidote-administration protocols; and drug-specific conservative treatment recommendations based on the available evidence or case reports. Documentation checklists and recommended contents for extravasation kits are also provided.

Delphi consensus for these drug-specific recommendations, including compress choice, antidote selection, and timing of intervention, was obtained at the level of the corresponding guideline section rather than separately for each individual recommendation.

## 5. Discussion

This study developed an evidence-based clinical guideline for the prevention, diagnosis, and management of non-chemotherapeutic drug extravasation, a complication affecting 0.1% to 6% of injections in clinical settings (1). Despite its frequency, this issue has received limited systematic attention. Existing protocols, such as the WOSCAN and NCCP guidelines, focus primarily on vesicant or chemotherapeutic agents. However, many non-chemotherapeutic drugs can also cause severe tissue damage or compartment syndrome, or may require surgical intervention.

Although some studies have addressed non-chemotherapeutic drugs, none have used a Delphi-based approach or been validated by clinical experts. The Delphi method strengthens the guideline’s validity and reliability, thereby enhancing its scientific and clinical value. Notable prior works include the 2020 guideline by Kim et al. and the systematic review by Ong and Van Gerpen (1, 27). This study is distinguished by its methodology and by addressing previously overlooked areas, including catheter selection based on patient-specific factors, a risk-based drug classification table, novel therapeutic recommendations, and diagnostic sensors for detecting extravasation before symptoms appear. These elements emerged during the Delphi process.

In contrast, studies by Reynolds et al., Shibata et al., and Le and Patel focus on injury mechanisms and initial treatments for noncytotoxic agents but omit critical aspects such as prevention techniques, diagnostic protocols, non-pharmacological interventions, extravasation kits, and documentation practices (8, 28, 29). This guideline introduces a multistep algorithm and reference tables linking drugs to compresses and antidotes.

### 5.1. Conclusions

This study addresses gaps in prior guidelines by presenting a protocol comprising 20 comprehensive sections on extravasation. It introduces four key innovations: 1) a drug risk-classification system to alert clinicians before injection, 2) recommendations for catheter selection to prevent incidents, 3) diagnostic sensors for early detection before visible symptoms, and 4) drug classification based on tissue-injury risk, with tailored initial therapeutic actions for each scenario.

Several items in the recommendation table lacked sufficient published data and received low-strength recommendations because of limited evidence. Although these recommendations were considered clinically acceptable by the expert panel, the available clinical evidence supporting interventions such as amniotic membrane, vacuum-assisted closure (VAC), hyperbaric oxygen therapy (HBO), and active Leptospermum honey remains limited.

The specialized nature of the topic posed challenges in recruiting experienced experts for the consensus process. However, conducting the study at Imam Hossein Hospital, a high-turnover referral center with a large patient volume and a structured extravasation documentation system, facilitated access to knowledgeable professionals. Two panel members participated in only one Delphi round because of scheduling conflicts. Given the specialized clinical focus of this guideline, the Delphi panel consisted of healthcare professionals with expertise in extravasation management. However, patient and caregiver perspectives were not sought through a separate consultation process, which may have limited the incorporation of patient values and preferences into the guideline recommendations. Future revisions could incorporate structured patient and caregiver feedback to complement expert consensus. In addition, the systematic review was restricted to English-language publications; therefore, relevant evidence published in other languages, including national guidelines, institutional protocols, and region-specific case series, may not have been captured. Although the panel comprised national experts, the guideline was developed through a systematic review of international literature and is designed to be adapted to local formularies and resources. Future multinational validation studies would further strengthen its global applicability. Implementing this guideline will require targeted training for care teams and regular updates based on new research. Overall, this guideline provides a structured, multidisciplinary framework for the prevention and management of non-chemotherapeutic extravasation and may serve as a practical resource for healthcare professionals until higher-quality implementation evidence becomes available.

ijpr-25-1-171391-s001.pdf

## Data Availability

All supporting materials for this guideline are included in the appendices submitted with the manuscript. No additional research dataset was generated or analyzed during this study.
