# Supplementary material for: Clinical Practice Guideline for the Management of Non-chemotherapeutic Extravasation: A Multidisciplinary Delphi-driven Consensus Study
Source: Iran J Pharm Res. 2026 Sep 9;25(1):e171391. doi: 10.5812/ijpr-171391 (PMC13620988; doi:10.5812/ijpr-171391)
Supplement: Supplementary file 1 [file ijpr-25-1-171391-s001.pdf]

Appendix A Guideline

Clinical Practice Guideline for the Management of Non-chemotherapeutic Extravasation, A Multidisciplinary Delphi-driven Consensus Study

(Running title: *Non-chemotherapeutic Extravasation Management*)

Clinical Practice Guideline for the Management of Non-chemotherapeutic Extravasation

1. **INTRODUCTION** **6**
    1.1 Objectives 6
    1.2 Educational Responsibility 6
2. **DEFINITIONS** **6**
    2.1 Extravasation 6
    2.2 Infiltration 6
    2.3 Erythema 6
    2.4 Phlebitis 6
    2.5 Conservative Management 6
    2.6 Impedance Sensor 6
    2.7 Swelling 6
        2.7.1 Measurement of Localized Swelling (X) 6
        2.7.2 Measurement of Arm Length (Y) 6
        2.7.3 Calculation of Swelling Percentage 6
    2.8 Drug Classification 7
    2.9 Catheter Classification 9
3. **SIGNS AND SYMPTOMS AT DIFFERENT LEVELS OF INJURY** **10**
    3.1 Symptom Classification 10
4. **MECHANISMS OF TISSUE INJURY** **12**
5. **CONSEQUENCES OF EXTRAVASATION** **14**
    5.1 Compartment Syndrome 14
6. **CLASSIFICATION OF DRUGS BASED ON RISK OF TISSUE INJURY** **15**
7. **RISK FACTORS FOR EXTRAVASATION** **18**
8. **PREVENTION** **19**
9. **SELECTION OF APPROPRIATE CATHETER** **20**
    9.1 For Drugs Compatible with Peripheral Administration 20
    9.2 For Drugs Incompatible with Peripheral Administration 20
    9.3 For Patients Requiring Repeated Phlebotomy 20
    9.4 Patients with Chronic Kidney Disease (CKD) 21
    9.5 Critically Ill Patients 21
    9.6 Special Populations 21
10. **DIAGNOSIS** **22**
11. **DETECTOR** **22**
    11.1 Impedance Sensing 22
    11.2 Temperature Sensors 22
    11.3 Radiofrequency (RF) Sensors 22
    11.4 Optical Sensors 22
    11.5 Ultrasound Sensors 22
    11.6 Fluid Pressure and Resistance 22
    11.7 Pressure–Skin Deformation Sensors 22
12. **DIFFERENTIAL DIAGNOSIS** **23**
    12.1 Venous Flare Reaction 23
    12.2 Vessel Irritation 23
    12.3 Venous Shock 23
    12.4 Phlebitis 23
    12.5 Cutaneous Hypersensitivity 23
13. **EXTRAVASATION KIT** **24**
14. **INITIAL THERAPEUTIC MANAGEMENT** **25**
    14.1 Nursing First-Response Checklist 25
15. **PHARMACOLOGIC MANAGEMENT** **26**
    15.1 Hyaluronidase 26
    15.2 Sodium Thiosulfate 26

15.3 Dimethyl Sulfoxide (DMSO) 26
15.4 Dexrazoxane 26
15.5 Phentolamine 26
15.6 Nitroglycerin 27
15.7 Terbutaline (B_2-agonist) 27
15.8 Antidotes with Uncertain Efficacy 27

16. NON-PHARMACOLOGICAL MANAGEMENT 28
16.1 Warm Compress 28
16.2 Cold Compress 28
16.3 Manual Massage or Pressure 28
16.4 Surgical Intervention 28
16.4.1 Salin flush out / Irrigation 28
16.4.2 Liposuction 29
16.4.3 Debridement and Skin Grafting 29

17. RECOMMENDED MANAGEMENT STRATEGIES FOR EACH DRUG 30

18. FOLLOW-UP 38

19. DOCUMENTATION AND PATIENT RECORDS 38

20. INVESTIGATIONAL TREATMENTS 38
20.1 Vacuum-Assisted Closure (VAC) 38
20.2 Hyperbaric Oxygen Therapy (HBO) 38
20.3 Amniotic Membrane 38
20.4 Active Leptospermum Honey (ALH) 39

21. REFERENCE 40

List of Tables

Appendix A table 1. Drug Classification Based on Tissue Injury Potential 8
Appendix A table 2. Venous Access Types and Characteristics 9
Appendix A table 3. Clinical Indicators and Symptoms of Drug Extravasation 10
Appendix A table 4. Severity Classification System for Extravasation-Related Tissue Injury 10
Appendix A table 5. Six Primary Mechanisms of Tissue Damage Following Drug Extravasation 12
Appendix A table 6. Non-Chemotherapeutic Drug Classification by Extravasation-Induced Tissue Injury Mechanism Following Extravasation 12
Appendix A table 7. Risk-Based Drug Classification with Initial Management Recommendations for Clinical Scenarios 15
Appendix A table 8. Risk Factor Categories for Extravasation Events 18
Appendix A table 9. Evidence-Based Prevention and Risk Mitigation Strategies 19
Appendix A table 10. Catheter Selection Criteria Based on Infusion Duration and Patient Factors 20
Appendix A table 11. Essential Components of Extravasation Emergency Response Kits 24
Appendix A table 12. Antidote Preparation and Administration Guidelines for Extravasation Management 27
Appendix A table 13. Conservative Treatment Protocols by Drug Classification 30
Appendix A table 14. Mandatory Documentation Requirements for Extravasation Incidents 38

List of Figure

Appendix A figure 1. Swelling Percentage Calculation Method for Injury Severity Assessment, *Image provided by Servier Medical Art (https://smart.servier.com/), licensed under CC BY 4.0 (https://creativecommons.org/licenses/by/4.0/).* 7
Appendix A figure 2. Standardized management protocol for extravasation events 25
Appendix A figure 3 Injection Sites for Hyaluronidase Administration**,** *Image provided by Servier Medical Art (https://smart.servier.com/), licensed under CC BY 4.0 (https://creativecommons.org/licenses/by/4.0/).* 26
Appendix A figure 4. Normal Saline Flush-Out Irrigation Procedure and Technique**,** *Image provided by Servier Medical Art (https://smart.servier.com/), licensed under CC BY 4.0 (https://creativecommons.org/licenses/by/4.0/).* 29

1. Introduction

1.1Objectives

- To describe key strategies for preventing drug extravasation.
- To identify diagnostic methods, tools, and early clinical signs for prompt detection of extravasation to limit tissue injury progression.
- To detail effective treatment options to reduce or prevent severe complications from extravasation.
- To highlight risk factors for extravasation to support closer monitoring and care for high-risk patients.
- To evaluate the extravasation risk of different drugs, encouraging caution during administration of high-risk agents.
- To provide guidance on selecting appropriate interventions after extravasation based on the type and extent of tissue damage.

1.2Educational Responsibility

- Healthcare administrators must ensure nurses and clinical staff receive proper training and demonstrate competence in managing extravasation, especially for vulnerable patients or those with known risk factors (1) .

2. Definitions

2.1 Extravasation

Extravasation occurs when a drug unintentionally leaks from a vein into surrounding tissue, a recognized complication of IV therapy (2). The severity depends on factors like the drug's physicochemical properties (e.g., osmolarity, pH), cytotoxicity, infusion rate, pressure, injection site characteristics, and patient-specific factors (3, 4).

Patients with malignancies or in critical care settings are at higher risk. Elderly individuals and neonates are particularly vulnerable due to their fragile, thin skin and subcutaneous tissue (5).

Leakage may result from direct vessel wall perforation by the needle or endothelial damage from the drug (2). Areas with thin skin, such as the dorsum of the hand, foot, or forearm, are common sites for injury. These locations, often used for IV administration, are prone to extravasation due to delicate skin and limited subcutaneous tissue (4).

Early signs include localized tingling, swelling, erythema, blistering, and pain. Visual inspection often underestimates underlying tissue damage, as deep necrosis and subdermal injury may extend beyond visible symptoms (2, 5).

2.2 Infiltration

Infiltration is the unintentional leakage of a non-vesicant drug or IV fluid into surrounding tissue. While similar to extravasation, infiltrated agents typically do not cause blistering or severe tissue damage due to their lack of vesicant properties (6).

2.3 Erythema

Erythema is an acute skin or mucosal reaction to drugs, marked by redness, rash, and irritation (7, 8).

2.4 Phlebitis

Phlebitis refers to vein inflammation at the cannula insertion site, most often linked to peripheral IV catheters (9, 10).

2.5 Conservative Management

Conservative treatment involves limb elevation, administration of antidotes like hyaluronidase, topical application of dimethyl sulfoxide (DMSO), and use of warm or cold compresses (11).

2.6 Impedance Sensor

Electrical impedance spectroscopy (EIS) is a non-invasive, cost-effective method to detect viable tissue and evaluate tissue integrity (12).

2.7 Swelling

- 2.7.1 Measurement of Localized Swelling (X)

To evaluate swelling at the injection or injury site, clinicians should measure the longest visible or palpable diameter using a ruler or measuring tape. This measurement is denoted as "X."

- 2.7.2 Measurement of Arm Length (Y)

To normalize swelling relative to patient body size, the length of the arm should be measured from the apex to the tip of the middle finger, regardless of the swelling location. This measurement is denoted as "Y."

- 2.7.3 Calculation of Swelling Percentage

The percentage of localized swelling is determined using the formula:

[Localized Swelling Percentage] = ([X]/[Y]) × 100

Where:

- X = Maximum length of the swollen area (cm)
- Y = Arm length from the axilla to the tip of the middle finger (cm)

This method offers a quick, standardized, and patient-specific way to quantify swelling. Normalizing to arm length allows for consistent comparisons across patients with varying body sizes (13).*[Figure 1]*

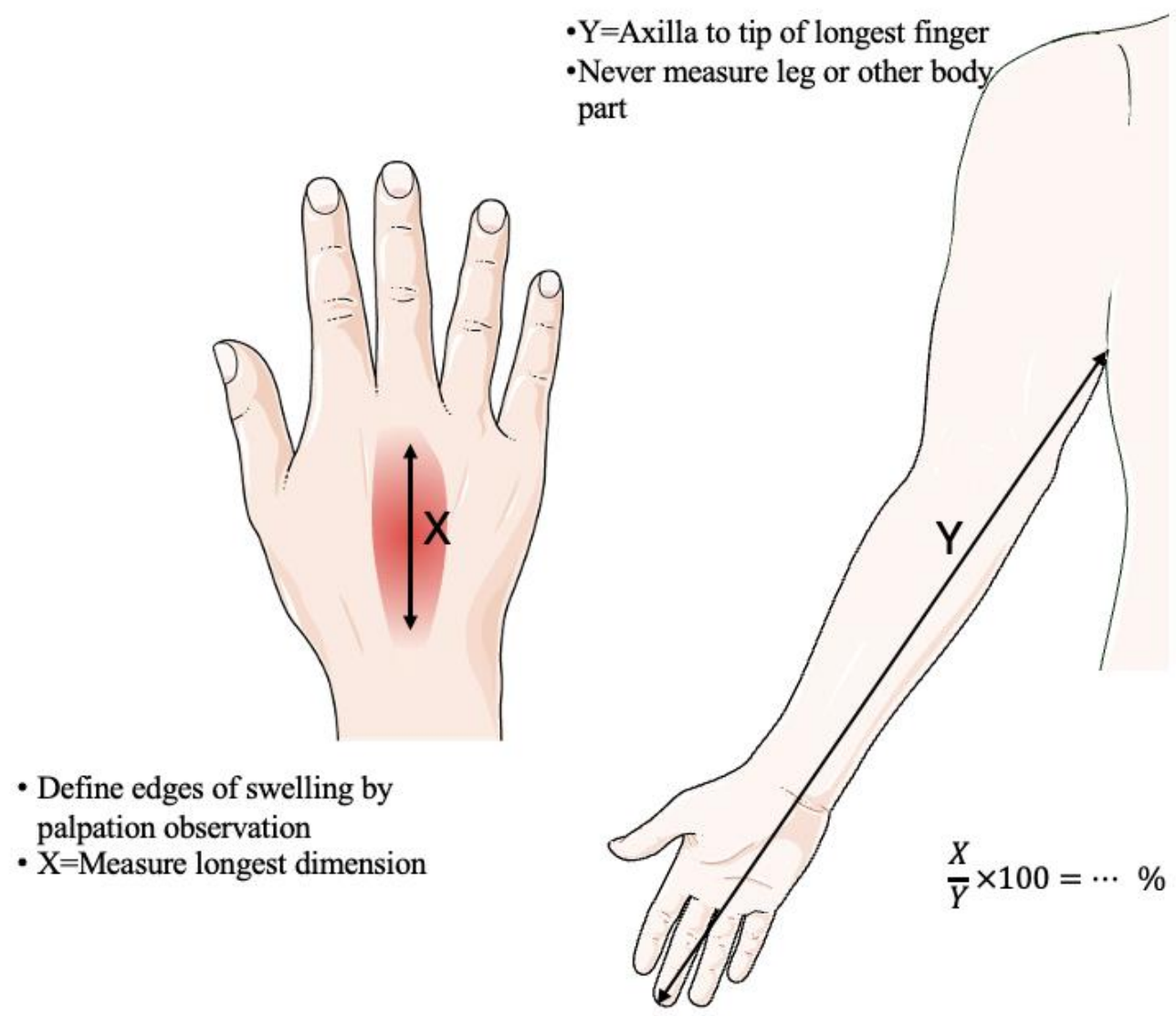

Appendix A figure 1. Swelling Percentage Calculation Method for Injury Severity Assessment, *Image provided by Servier Medical Art (https://smart.servier.com/), licensed under CC BY 4.0 (https://creativecommons.org/licenses/by/4.0/).*

2.8 Drug Classification

Intravenous medications are divided into five categories based on the potential severity of tissue injury from extravasation: vesicants, exfoliants, irritants, inflammatants, and neutral agents (14, 15).*[Table 1]* However, some drugs may not fit neatly into one category, and their local effects can differ significantly from other agents within the same group. This is especially relevant for patients receiving multiple medications through a single IV line. Furthermore, the extent of tissue damage caused by certain agents may depend on their concentration and duration of exposure (15).

Appendix A table 1. Drug Classification Based on Tissue Injury Potential

**Vesicant**	Vesicants are drugs that, when extravasated, may cause blistering, inflammation, severe tissue necrosis, and pain. This category includes chemotherapeutic agents, vasoconstrictors, parenteral nutrition solutions, hyperosmolar agents, and acidic or alkaline preparations (pH < 5 or > 9) (16). Examples: Aminophylline, Acyclovir, Metronidazole, Penicillin, Valproate, and others (17).
**Exfoliant agent**	These drugs cause local inflammation and skin desquamation. Some may have limited vesicant potential, though they are not strictly classified as vesicants (15).
**Irritants**	These drugs cause pain, burning, or discomfort during IV administration but generally do not result in blistering. Minor extravasation typically leads to localized, short-term effects without deep tissue damage (15, 16). Small-volume extravasation of these agents usually causes limited, localized tissue injury with minimal spread (16). Examples: Nafcillin, Potassium Phosphate, Potassium Chloride, Vancomycin, and others (17).
**Inflammatory agent**	These agents trigger mild to moderate localized inflammation at the extravasation site, sometimes with erythema, but typically without significant pain (15). Examples: Calcium Chloride, Sodium Bicarbonate, Nicardipine, and others (18).
**Neutrals**	These drugs generally do not cause inflammation or tissue damage when extravasated (15).
Reference: (15)	

2.9 Catheter Classification

[Table 2] outlines the classification and main attributes of venous access devices.

Appendix A table 2. Venous Access Types and Characteristics

**Peripheral IV Catheter**	This catheter, ranging from 3 to 6 cm in length, is typically inserted into superficial peripheral veins via the antecubital fossa. Like ultrasound-guided peripheral IV catheters, midline catheters, and non-tunneled central venous catheters, it features a polyethylene or silicone flange that secures the catheter within the subcutaneous tissue. This design also serves as a barrier, preventing bacterial migration along the device's external surface.	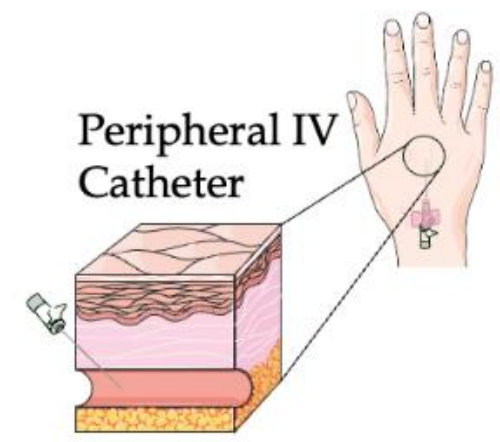
**Ultrasound-Guided Peripheral IV Catheter**	Inserted into deeper arm veins when superficial veins are not visible or palpable, this catheter enables the placement of long peripheral IV catheters (longer than 8 cm).	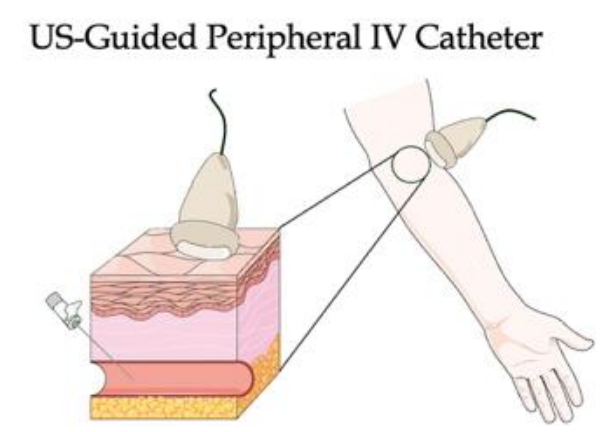
**Midline Catheter**	Midline catheters, measuring 7.5 to 25 cm in length, are usually inserted into veins above the antecubital fossa, with the catheter tip placed in the basilic or cephalic vein, extending toward the subclavian vein.	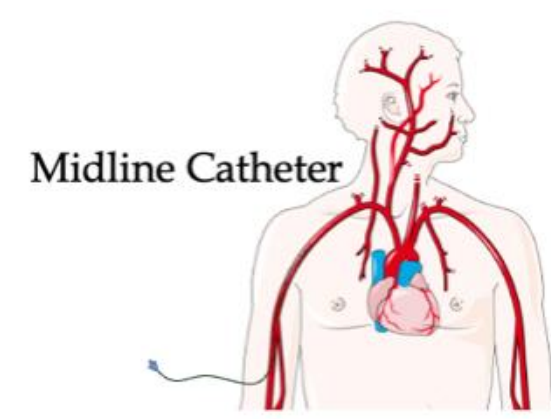
**Non-Tunneled Central Venous Catheter**	These short-term central venous catheters, ranging from 15 to 25 cm in length, are typically used for 7 to 14 days. They are placed through direct venous puncture and cannulation of large central veins, such as the internal jugular, subclavian, or femoral veins.	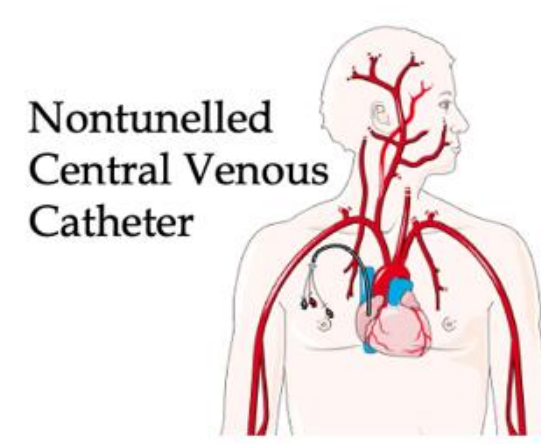
**Tunneled Central Venous Catheter**	This catheter features a subcutaneous tunnel between the insertion site and the venous access point, which lowers the risk of bloodstream infections by reducing direct bacterial migration from the skin.	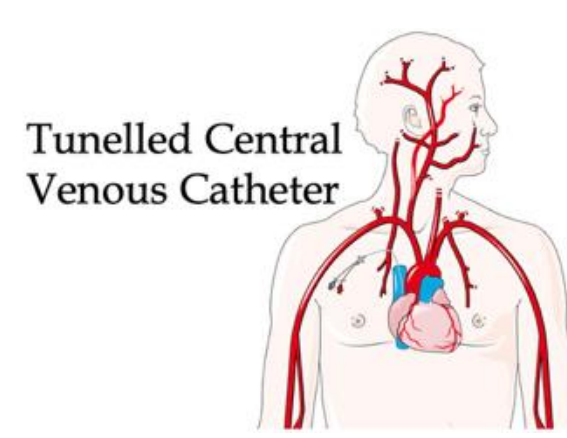
**Implanted Port**	This central venous access device, implanted subcutaneously in the chest, consists of a reservoir for injection connected to a catheter that ends in a deep chest vein. Implanted ports are preferred for their long-term durability and better cosmetic appearance.	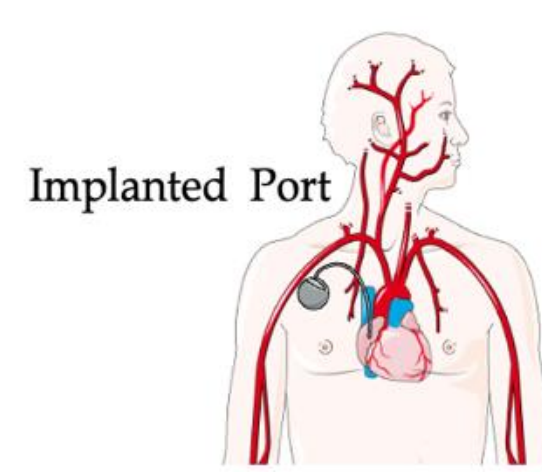

**Arterial Catheter**	This catheter, inserted into an artery, is mainly used in intensive care settings for continuous blood pressure monitoring and arterial blood gas analysis. The radial artery at the wrist is the preferred site due to its accessibility and low complication rate (19).	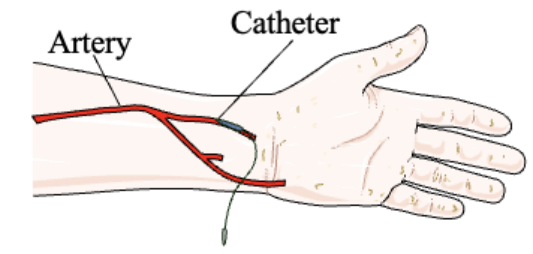 Arterial Catheter
**Peripherally Inserted Central Catheter (PICC Line)**	This catheter, typically longer than 45 cm, is used for long-term vascular access. In adults, it is inserted through peripheral arm veins and advanced until the catheter tip is positioned in the lower superior vena cava or upper right atrium. These catheters offer central venous access comparable to other central lines but avoid the risks of direct puncture of deep veins in the neck, chest, or groin.	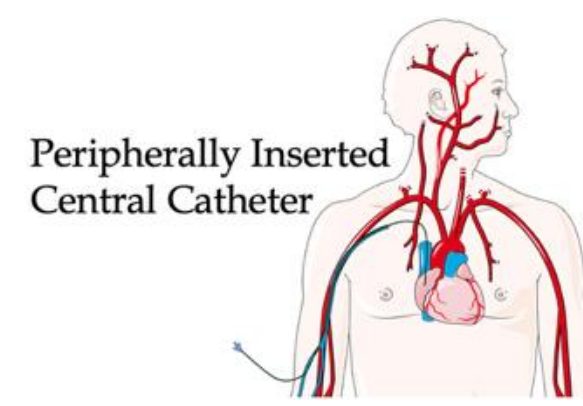
Reference: (20) , *Image provided by Servier Medical Art (https://smart.servier.com/), licensed under CC BY 4.0 (https://creativecommons.org/licenses/by/4.0/).*		

3. Signs and Symptoms at Different Levels of Injury

Extravasation can lead to tissue damage and complications ranging from mild to severe, including desquamation and tissue necrosis. Early symptoms, appearing soon after extravasation, include burning, tingling, pain, itching, swelling, edema, induration, and blistering. If untreated, symptoms may worsen within 2 to 3 days, progressing to ulceration, erythema, tissue induration, desquamation, and necrosis (15, 21).*[Table 3]* Delayed treatment can result in more extensive damage to surrounding structures, such as joints, nerves, and tendons (15).

Appendix A table 3. Clinical Indicators and Symptoms of Drug Extravasation

Scarring (24)	Blistering (24)	Necrosis (23)	Inflammation (23)	Redness (23)	Burning sensation (23)	Pain (22)
Induration (22)	Discomfort (22)	Phlebitis (22)	Tingling (23)			
Pallor at the site (22)	Loss of function of the affected limb(23)	Tenderness (22)				
Swelling and edema (22)	Changes in temperature at the infusion site (23)	Feeling of pressure (22)				
Resistance to infusion and decreased flow rate (22)						
Leakage around the catheter, needle, or implanted port (22)						

As noted earlier, extravasation from central or peripheral catheters often presents with symptoms such as redness, edema, pain, numbness, pruritus, drug leakage around the catheter site, impaired blood return, or reduced or stopped infusion flow. These signs may appear immediately or shortly after drug administration (23). Patients should promptly report any unusual sensations to the healthcare team to support early diagnosis. Consequently, it is critical for both healthcare providers and patients to understand these symptoms and their clinical significance (23). Extravasation symptoms are categorized into five severity grades (17).

3.1 Symptom Classification

The severity grading system for tissue injury related to extravasation is presented in *[Table 4]*.

Appendix A table 4. Severity Classification System for Extravasation-Related Tissue Injury

**Grade**	0	1	2	3	4
**Skin Color**	Normal skin tone (23)	Mild pink discoloration (23)	Noticeable redness (23)	Brick-red skin, possibly surrounded by pale areas (25 ,23)	Blackened skin with surrounding pallor (25 ,23)
**Tissue Injury**	Intact, undamaged skin (23)	Presence of blisters (23)	Blistering with skin peeling (23)	Exposure of subcutaneous tissue (23)	Muscle or bone exposure, with tissue necrosis (23)

**Pain**	Mild pain (25)	Mild to moderate pain (25)	Moderate to severe or no pain (due to nerve damage) (25)	No pain due to nerve damage	No pain due to nerve damage
**Mobility**	Full mobility (23)	Slight limitation (23)	Moderate limitation (23)	Immobility (23)	Complete immobility (23)
**Swelling**	None (25)	Minimal (1–10%) (25)	Mild (<15%) (25)	Moderate (25–50%) (25)	Severe (>50%) (25)
**Tissue Temperature**	Normal (23)	Warm (23)	Warm (23)	Cool (25)	Cool (25)
**Perfusion and Pulse**	Normal perfusion and pulse (25)	Normal perfusion and pulse (25)	Normal capillary refill (1–2 sec) (17), normal pulse (25)	Normal or reduced perfusion; capillary refill time normal or slightly delayed(17), Normal pulse (25)	Diminished perfusion, prolonged capillary refill (>4 sec) (17) reduced pulse(25)

4. Mechanisms of Tissue Injury

In some instances, understanding the mechanism of tissue injury is essential for choosing effective management strategies (25). The pathophysiology of extravasation-related injury depends on factors such as the drug's cytotoxicity, pH, osmolarity, vasoconstrictive or vasodilatory effects, the volume of extravasated fluid, and the duration of tissue exposure. For instance, vasoconstriction or large infusion volumes can lead to pressure-related mechanical injuries (16).*[Table 5]* However, tissue damage typically results from a combination of multiple mechanisms rather than a single factor (26).

Appendix A table 5. Six Primary Mechanisms of Tissue Damage Following Drug Extravasation

**Mechanisms of Tissue Injury**	
**1**	Direct tissue cytotoxicity
**2**	Injury caused by vasoconstriction induced by the drug
**3**	Damage due to osmotic pressure
**4**	Mechanical compression from extravasated fluid volume
**5**	pH-related injury
**6**	Poor tissue absorption and prolonged exposure
Reference: (16)	

1) Although often linked to chemotherapeutic agents, cytotoxicity also occurs in some non-chemotherapy drugs. These agents can show carcinogenic, mutagenic (genotoxic), and organotoxic effects even at low concentrations (14).
2) Strong vasoconstriction in capillary smooth muscle greatly reduces blood flow, which results in ischemia and tissue necrosis. High vasopressor concentrations at the infiltration site activate alpha-adrenergic receptors. This action increases vascular resistance, raises interstitial pressure, and drives extravasation into surrounding tissues. Consequently, ischemia may worsen, and severe cases can lead to compartment syndrome (16, 25).
3) Any shift from the normal body fluid osmolality of about 310 mOsm/kg, whether hyperosmolar or hypoosmolar, irritates the vascular wall, damages cellular proteins, and causes tissue injury and cell death (25).
In hypotonic solutions below 200 mOsm/kg, especially under 112 mOsm/kg, excess fluid enters cells and leads to rupture. Hypertonic drugs generally cause more frequent and severe damage than hypotonic ones. Injuries begin above 500 mOsm/kg and become marked above 900 mOsm/kg. Such damage continues until the solution reaches isotonicity. Even small extravasations of hypertonic drugs can result in serious injury. In these situations, fluid moves from cells to the extracellular space, leading to apoptosis and cell death through reactive oxygen species (ROS) formation, DNA and protein damage, and altered cell membrane permeability. The osmolality of the extravasated solution directly relates to the severity of tissue injury (16, 18, 27, 28).
4) At times, the volume of extravasated drug disrupts tissue perfusion by increasing hydrostatic pressure. Separate from the drug's physicochemical properties, this mechanical compression alone can cause compartment syndrome and permanent tissue damage (18).
5) Solutions with a pH near the physiological level of 7.4 are generally safe for injection. However, when pH falls outside the 5 to 9 range, risks increase for endothelial damage, vasoconstriction, inflammation, edema, desquamation, ulceration, and vessel rupture. The degree of deviation corresponds directly to injury severity. Solutions well beyond this range can lead to major tissue damage (25, 28). Acidic solutions primarily cause coagulative necrosis and scar formation. In contrast, alkaline solutions degrade proteins and collagen in the extracellular space. Alkaline extravasations are usually more severe, reach deeper tissues, and persist in causing damage until fully neutralized, mainly because of hydroxyl ion production (18, 25, 29).
6) The extent of tissue damage depends on the duration of drug-tissue contact. Poorly absorbed or insoluble agents stay in the tissue longer, prolonging exposure and worsening injury (16).
[Table 6] summarizes non-chemotherapeutic drugs grouped by their potential tissue damage following extravasation.

Appendix A table 6. Non-Chemotherapeutic Drug Classification by Extravasation-Induced Tissue Injury Mechanism Following Extravasation

**Drugs Categorized by Mechanism of Extravasation-Induced Tissue Damage**					
**Concentrated electrolyte**	**Hyperosmolar**	**Mechanical Compression**	**Basic or Acidic**	**Vasoconstrictor**	**Direct cellular toxicity**
• calcium chloride (18)<br>• calcium gluconate (10%) (18)<br>• magnesium sulfate (18)	• aminophylline: 170 mOsm/L(25)<br>• ampicillin50mg/mL: 566 mOsm/kg (25)	• propofol (18)<br>• soybean oil (INTRALIPOS®) (18)	• acyclovir (25)<br>• arginine (25)<br>• Aminophylline (30)<br>• Amiodarone (25)<br>• amphotericin (30)	• adrenaline (18)<br>• dobutamine (25)<br>• dopamine (25)<br>• epinephrine (25)	• Diazepam (Excipient: propylene glycol)(18)<br>• digoxin(Excipient: propylene glycol) (18)

• potassium chloride (18)<br>• saccharated ferric oxide (Fe2+)(18)<br>• sodium bicarbonate (18)<br>• sodium chloride (18)	• calcium chloride 10% (25)<br>• calcium gluconate or calcium gluceptate 680mOsm/L (25)<br>• contrast media (29)<br>• digoxin (25)<br>• diazepam>2000 mOsm/kg (25)<br>• glucose >10% (18)<br>• Iopamidol (18)<br>• magnesium sulphate 20% (30)<br>• mannitol>5% (18)<br>• parenteral nutrition>900 mOsm/L (25)<br>• potassium chloride>40 mEq/L (25)<br>• sodium bicarbonate ≥8.4% (25)<br>• sodium chloride>1% (25)<br>• etomidate (25)<br>• lorazepam: >2000 mOsm/kg (25)<br>• mannitol ≥20% (25)<br>• nafcillin40mg/mL:402 mOsm/kg (25)<br>• sodium phosphate: 7000 mOsm/L (25)		•ampicillin sodium (18)<br>•cloxacillin sodium hydrate (MCIPC) (18)<br>•conivaptan (25)<br>•dantrolene (25)<br>•doxycycline (25)<br>•diazepam (30)<br>•Erythromycin (30)<br>•Esmolol (25)<br>•Furosemide (18)<br>•Gentamicin (25)<br>•hydroxyzine hydrochloride (18)<br>•lansoprazole (18)<br>•nafamostat mesylate (18)<br>•nicardipine hydrochloride (18)<br>•phenobarbital (25)<br>•pentobarbital (25)<br>•phenytoin (25)<br>•potassium canrenoate (18)<br>•promethazine (25)<br>•thiopental (30)<br>•vancomycin (25)<br>•immune globulin (25)<br>•pentamidine (25) omeprazole (18)	• methylene blue (25)<br>• noradrenaline (18)<br>• norepinephrine (25)<br>• phenylephrine (25) vasopressin (25)	• gabexate mesylate(18)<br>• hemin(Excipient: propylene glycol)(18)<br>• sulfamethoxazole (SMX) (18)<br>• trimethoprim (TMP) (18)

5. Consequences of Extravasation

Delayed recognition and diagnosis of extravasation can result in severe, sometimes irreversible complications, including tissue necrosis, tendon injury, and damage to nerves and joints. These issues can significantly affect a patient's physical function and appearance, potentially causing stress, psychological trauma, and emotional distress (11, 26, 30, 31). Given these physical and emotional challenges, compassionate care and support from medical staff are vital to help restore patient function and encourage treatment adherence (31).

Other complications from tissue injury include skin infections with abscess formation, inflammation, necrotizing fasciitis, and progressive functional impairment or mobility limitations due to pain (30-32). These can severely impact a patient's social functioning and quality of life. In some cases, pain may become so severe that opioid analgesics are necessary for relief (23).

In advanced or late-diagnosed cases, outcomes may include loss of skin integrity, muscle contracture, complete loss of limb function, or the need for surgical intervention (11, 33). Additionally, extravasation can extend hospital stays, increase medical costs, require reconstructive surgeries, strain the patient-provider relationship due to perceived negligence, and delay treatment of the underlying condition (23, 26, 30).

Extravasation is also a recognized cause of compartment syndrome:

5.1 Compartment Syndrome

Compartment syndrome is rare, but failure to treat it within the first 6 to 8 hours can lead to severe and irreversible damage (34).

This syndrome may arise from several causes, such as trauma, burns, infections, insect or animal bites, excessive muscle exertion, hemorrhage, and extravasation (34).

When fluid volume increases within a muscle compartment bounded by inelastic fascia, internal pressure rises. This elevated pressure compresses arterial and venous vessels, lowers tissue perfusion, and damages muscles and nerves (35). Further pressure increases can cause complete neural blockade and motor paralysis (36), while ongoing ischemia results in cell death and myocyte destruction (37).

Compartment syndrome has an early phase within the first 4 hours of elevated pressure, followed by a late phase marked by muscle destruction (37). The key difference between these phases is the level of muscle damage, as longer and more intense ischemia raises the risk of myonecrosis (37).

Extravasation-induced Compartment Syndrome:

The placement of an intravenous (IV) catheter plays a significant role in the risk of extravasation and subsequent compartment syndrome (38). High-pressure infusion through a peripheral venous catheter is often the main trigger (39).

Clinical signs, such as swelling on palpation, pain, pallor, paresthesia, and pulselessness, typically appear in later stages. By the time these signs are noticeable, irreversible tissue damage has often already occurred (40). To avoid the severe outcomes of delayed treatment, emergency fasciotomy may be required (40).

As previously noted, the primary mechanism of compartment syndrome involves increased compartmental content, which raises interstitial fluid pressure, collapses capillaries, reduces local perfusion, and leads to cell death, neurovascular compromise, and muscle injury (34).

Although intra-compartmental pressure measurement can aid in diagnosis, current devices often lack precision. Consequently, clinical judgment remains the primary diagnostic tool, especially in critically ill or unconscious patients who cannot report symptoms. Regular and thorough examination of the affected limb is therefore critical for early detection and reducing the risk of permanent damage (34).

6. Classification of Drugs Based on Risk of Tissue Injury

Initial Management Recommendations According to Drug Risk Level are summarized in *[Table 7]*.

Appendix A table 7. Risk-Based Drug Classification with Initial Management Recommendations for Clinical Scenarios

Risk of Extravasation	drug	Initial Recommended Management
**Low risk**	Acetylcysteine	• For localized swelling exceeding 30%, conduct a clinical evaluation within 30 minutes. If extravasation is confirmed, monitor hourly for 48 hours. Typically, intensive intervention is unnecessary unless tissue ischemia or swelling beyond 60% is observed. Regular monitoring aims to identify injury progression, prompting a plastic surgery consultation and prompt therapeutic action. In these cases, hyaluronidase administration is generally advised.<br>• For swelling below 30% due to extravasation of agents in this category, urgent intervention is generally not required. Instead, perform clinical assessments every 4 hours for 24 hours. Treatment is typically unnecessary unless tissue ischemia or increasing swelling develops, at which point site irrigation and potential medical management may be needed.
	Aminophylline	
	Amphotericin B liposomal	
	Ampicillin	
	Benzylpenicillin	
	Cefazolin	
	Cefotaxim	
	Ceftazidime	
	Ceftriaxone	
	Cefuroxime	
	Clindamycin	
	Fentanyl	
	fluconazole	
	Flumazenil	
	Fosphenytoin	
	Furosemide	
	Gentamicin	
	10%> Glucose	
	Haloperidol	
	Heparin	
	Imipenem/Cilastatin	
	Indometacin	
	Iron infusion	
	IVIG	
	Labetalol	
	Lacosamide	
	Lactated ringers	
	Lidocaine	
	Linezolide	
	Magnesium	
	Magnesium Sulfate	
	Meropenem	
	Methylprednisolone	
	Nimodipine	
	Omeprazole	
	Pamidronate	
	Pantoprazol	
	Pentamidine	
	Piperacillin/Tazobactam	

	Rifampin	
	Sodium Chlorid<3%	
	Tobramycin	
**Intermediate risk**	Acetazolamide	
	Alteplase	
	Amikacin	
	Any medication containing propylene glycol	
	Arginine	
	Atropin	
	Ciprofloxacin	
	Clinazepam	
	Cyclizine	
	Dantrolene	
	Diazepam	
	Digoxin	
	Diphenhydramin	
	Droperidol	
	Erythromycin	
	Erythromycin	
	Etomidate	
	Flucloxacillin	
	Ganciclovir	
	Glucose10 to 12.5%	
	Hydralazine	
	Lorazepam	
	Midazolam	
	Milrinone	
	Morphine	
	Mycophenolate	
	Neostigmin	
	Nitroglycerine	
	Ondansetron	
	Phenobarbital	
	Phenytoin	
	Propofol	
	Protamin	
	Quinine	
	Radiographic contrasts	
	Sulfamethoxazole	
	Thiopental sodium	
	trimethoprim	
	Vancomycin	

	Zink	
**High risk**	Aciclovir	• When vasoactive agents cause extravasation, quickly assess the site, administer phentolamine, and avoid using cold compresses.<br>• For extravasation causing swelling over 30%, complete a clinical evaluation within 30 minutes and begin site irrigation within 12 hours of injury identification.<br>• For swelling below 30%, conduct an initial clinical assessment within 30 minutes, then monitor hourly for 48 hours.<br>In such cases, irrigation is indicated only if unexpected tissue ischemia occurs.
	Alprostadil	
	Amiodarone	
	Ascorbic acid	
	Caffeine citrate	
	Calcium (all salt forms)	
	Diazixide	
	Doxycycline	
	Epoprostenol	
	Esmolol	
	Foscarnet	
	12.5%< Glucose	
	Iron	
	Isoprenaline	
	Mannitol	
	Nitroprusside	
	Potassium chloride>40 mmol/L	
	Promethazine	
	3% ≤ Sodium bicarbonate	
	3% ≤ Sodium chloride	
	Total parental nutrition (TPN)	
	Vasoactive medication (eg ,adrenaline, dobutamine, dopamine ,milrinone, noradrenaline ,phenylephrine, prostaglandins (vasopressin	
Reference:(41)		

7. Risk Factors for Extravasation

Extravasation depends on multiple factors. These include patient-specific anatomical traits, such as vein size, fragility, and comorbidities, as well as the drug's physicochemical properties. The duration of tissue exposure, the volume of direct infiltration, and mechanical factors—like venipuncture technique, catheter type, number of cannulation attempts, and the injector's skill and experience—also play a role (42).*[Table 8]*

Appendix A table 8. Risk Factor Categories for Extravasation Events

**Risk Factors for Extravasation**		
**Infusion-Related Factors**	• Poor catheter or dressing fixation (23)<br>• CVAD malfunction (14)<br>• Incorrect CVAD placement site (14)	• Prolonged infusion duration (14)<br>• High injection speed or pressure (23)<br>• Inappropriate equipment, such as incorrect needle or catheter size (14)<br>• Wet or inadequate dressing (23)
**Drug-Related Factors**	• Osmolarity of the infused drug (21)<br>• Cytotoxicity (21)<br>• Volume of extravasated drug (21)	• High drug volume or concentration (14)<br>• pH of the infused drug (43)
**Patient-Related Factors**	• Impaired pain perception (e.g., due to sedation, intubation, neuropathy, or coma) (21)<br>• Obesity or poor vein access (23)<br>• Peripheral vascular disorders (e.g., diabetes, Raynaud's, lymphedema) (21)<br>• Endothelial dysfunction (e.g., in elderly patients, diabetics, or those with atherosclerosis, hypertension, dyslipidemia, or hepatic failure) (21)	• Age (pediatric or elderly patients) (23)<br>• Immunocompromised status (21)<br>• Fragile or small veins (23)<br>• Injection into unsuitable sites, such as joints, tendons, distal lower limb veins, or dorsal hand veins (21)<br>• Damaged skin (e.g., eczema, psoriasis, scars, radiation dermatitis) (43)<br>• High patient mobility (23)
**Healthcare Personnel Factors**	• Multiple cannulation attempts(23)	• Lack of experience (23)<br>• Inattention or failure to inspect the site during dressing changes (21)

8. Prevention

Preventing extravasation is far more effective than managing its outcomes. The first step in prevention is identifying and addressing modifiable risk factors (21, 44). Among the many risk factors, incorrect administration technique stands out as a leading cause of extravasation. For this reason, focused staff training, increased awareness, and strict adherence to standardized protocols are critical preventive measures (21, 44, 45).*[Table 9]* By adopting evidence-based management protocols, most extravasation incidents can be avoided (23).

Regular monitoring and palpation, especially in high-risk groups like neonates, elderly patients, and those who are unconscious, are key preventive strategies. Any suspected extravasation should be treated as a medical emergency (11, 21)

Appendix A table 9. Evidence-Based Prevention and Risk Mitigation Strategies

Healthcare Personnel Responsibilities	Patient-Centered Interventions	Injection Method, Site & Equipment
• Stop infusion immediately if extravasation is suspected (23)<br>• Ensure staff have appropriate knowledge and skills (23)<br>• Avoid distractions and ensure organized drug preparation (23)<br>• Confirm IV patency before and during infusion (23)<br>• Flush between medications (23)<br>• Routinely inspect the site for swelling or erythema (23)<br>• Engage with patients about any discomfort (23)<br>• Know the components and site of IV kits (23)	• Inform patient about warning signs and risks (46)<br>• Educate patient on warning signs and risks (31)<br>• Encourage prompt reporting of pain, burning, or leakage (31)	• Select optimal peripheral access sites (e.g., dorsal hand, antecubital vein) (23)<br>• Use appropriately sized cannulas/needles (31)<br>• Choose longer needles for obese patients (31)<br>• Avoid long-acting anesthetics before cannulation (31)<br>• Alternate limbs for prolonged infusions (31)<br>• Secure catheter firmly with transparent dressing (23)<br>• Regulate infusion pump pressure (23)<br>• Create a new access point for vesicant drugs (23)<br>• Dilute irritants when appropriate (23)<br>• Elevate limb post-extravasation (depending on injury mechanism) (23)<br>• Verify blood return before infusion (23)<br>• Prefer central venous access where indicated (46)<br>• Flush with saline before medication administration (23)<br>• Limit PIV dwell time to ≤24 hours (46)<br>• Ensure proper septum needle placement in central access (46)<br>• Maintain hand and site hygiene<br>• Avoid high-speed infusion

Key prevention strategies include:

1. Choosing suitable veins (avoiding small, fragile, or joint-adjacent veins)
2. Reducing repeated venipuncture and trauma
3. Selecting an appropriately sized plastic cannula
4. Ensuring the needle entry site remains visible
5. Administering vesicants first in multi-drug regimens
6. Verifying blood return before starting infusion
7. Opting for central venous access for prolonged infusions (47)

9. Selection of Appropriate Catheter

9.1 For Drugs Compatible with Peripheral Administration

- < 5 days duration:

Peripheral IV catheter or ultrasound-guided peripheral intravenous (PIV) is recommended. Peripherally inserted central catheters (PICC) are not suitable for such short-term use (20).

- 6–14 days:

PICC is an option, but ultrasound-guided PIV or midline catheters are preferred due to lower complication rates (20), In critically ill patients, non-tunneled central venous catheters (CVCs) are prioritized when inserted by experienced personnel (20).

- 15–29 days:

PICC is generally preferred over midline catheters. However, midlines may be used in select cases for up to 4 weeks 48-) (50.

- >30 days:

Tunneled CVCs or implanted ports are indicated. These are reserved for patients with difficult venous access, venous thrombosis, or those requiring intermittent infusions over an extended period (20).

9.2 For Drugs Incompatible with Peripheral Administration

- Vesicants and irritants

PICC is acceptable for all durations.

- ≤14 days:

A non-tunneled CVC may be used if inserted by experienced personnel.

- ≥15 days:

Tunneled or cuffed catheters are appropriate for prolonged administration of vesicants (20).

9.3 For Patients Requiring Repeated Phlebotomy[1]

- 6–14 days:

For patients needing frequent phlebotomy (>3/day) or those with difficult venous access requiring >6 days of infusion, PICC, non-tunneled CVC, ultrasound-guided PIV, or midline catheters are viable options. However, midline and ultrasound-guided PIV are preferred for this duration (20).

- 14–29 days:

For prolonged administration of vesicants, tunneled catheters are advised.

- >30 days:

When long-term access is needed and venous access is challenging, tunneled central catheters are the optimal choice (20). In *[Table 10]*, we briefly present recommendations for catheter selection.

Appendix A table 10. Catheter Selection Criteria Based on Infusion Duration and Patient Factors

**Infusion Duration**	**Compatible Drugs for Peripheral Administration**	**Incompatible Drugs for Peripheral Administration**	**Difficult Venous Access**	**Repeated Phlebotomy**
≤ 5 days	Peripheral IV catheter or US-guided peripheral IV catheter	PICC	In difficult access: US-guided peripheral IV catheter > Peripheral IV catheter	Peripheral IV catheter or US-guided peripheral IV catheter
6–14 days	US-guided peripheral IV catheter or Midline catheter. In critically ill patients: Non-tunneled Central Venous Catheter (CVC)	PICC<br>In critically ill patients with hemodynamic monitoring needs: Non_tunneled CVC >PICC	US-guided peripheral IV catheter > Peripheral IV catheter<br>In critically ill patients: Midline catheter or Non-tunneled CVC > PICC	Midline catheter or Non-tunneled CVC > PICC
15–30 days	PICC > Midline catheter	PICC or Tunneled CVC or Implanted Port	PICC > Tunneled CVC	PICC > Tunneled CVC
> 31 days	PICC > Implanted Port or Tunneled CVC	PICC or Tunneled CVC or Implanted Port	Implanted Port or PICC	Tunneled CVC or PICC

[1] The process of venipuncture for blood sampling, blood donation, transfusion, and other related procedures.

Reference: (20)

9.4 Patients with Chronic Kidney Disease (CKD)

Patients with CKD stages A1 to A3 (estimated glomerular filtration rate [eGFR] > 45 mL/min) have the same indications for PICC use as those without major renal impairment. Still, if the degree of renal dysfunction remains unclear, consult a nephrologist before PICC insertion (20, 51-55).

For patients with CKD stage B3 or higher (eGFR < 45 mL/min), saving peripheral and central veins takes precedence to support possible future hemodialysis or arteriovenous access (fistulas or grafts). As a result, PICCs and midline catheters should not go into the arm veins of these patients, no matter the indication (20, 55).

- < 5 days:

For infusions lasting less than 5 days with compatible drugs, place peripheral IV lines in the dorsum of the hand, steering clear of antecubital veins (56).

- 6–14 days:

For infusions lasting 6 to 14 days or involving incompatible drugs, choose small-bore tunneled central catheters, such as 4 French single-lumen or 5 French double-lumen catheters inserted in the internal jugular vein (56).

In dialysis patients, discuss drug administration timing with a nephrologist, particularly for delivery during or after dialysis sessions. However, for patients with CKD stage B3 or higher, base decisions on individual factors, including urgency, vascular preservation, dialysis likelihood, and available institutional resources (20)

9.5 Critically Ill Patients

- < 5 days:

Peripheral IV or midline catheters are suitable for durations under 5 days (20, 57).

- 6–14 days:

Peripheral IV and midline catheters remain appropriate. However, under specific clinical conditions, CVCs may be preferred (20).

- > 15 days:

For treatments exceeding 15 days, particularly in critically ill patients with coagulopathies, PICC lines are typically preferred. Yet, if the patient is hemodynamically unstable or receiving vasopressors, prioritize CVCs. In coagulopathic patients needing long-term access, PICCs are generally the first choice (20).

9.6 Special Populations

- Patients with ≤ 5 hospital admissions per year:

For compatible drug infusions, use a midline catheter for 6–14 days, and opt for a PICC for durations ≥15 days (20). Patients with infrequent hospitalizations are not suitable candidates for tunneled or cuffed catheters or implanted ports.

- Patients with ≥ 6 hospital admissions per year:

Peripheral IV catheters are appropriate for up to 5 days. For 6–14 days, midline catheters are preferred. For durations ≥15 days, tunneled or cuffed central venous catheters are recommended, with implanted ports considered optimal for ≥31 days (20). For repeated phlebotomy or infusion of non-vesicant, non-irritant drugs lasting more than 15 days, PICCs are also recommended. Regardless of duration, PICCs are appropriate for administering irritant or vesicant drugs (20).

10. Diagnosis

Extravasation is mainly diagnosed by visual inspection and spotting general signs, including pain, edema, erythema, discoloration, and swelling at the injection site. For this reason, nursing personnel carry the primary duty for early detection (58, 59).

In addition, specific signs consist of no blood return, odd resistance when aspirating with a syringe, and halted infusion (26).

When detection occurs late, serious tissue injury and permanent problems may arise, harming both function and appearance. This outcome highlights the value of prompt recognition (26).

During infusion, clinicians can confirm needle placement in the vessel and exclude extravasation by giving a small dose of intravenous sodium bicarbonate. With correct placement, CO_2 from the dose appears in the patient's exhaled air, indicating proper positioning or extravasation (60).

11. Detector

A main sign of correct cannula placement is the clear pressure change felt when the needle pierces the vein's wall.

Extravasation detection in its early stages often relies on outside signs that lack specificity. This touch feedback, as well as visual checks, depends strongly on the injector's skill. For that reason, sensing technologies help greatly in verifying proper catheter placement and in spotting extravasation right away.

- Unimodal Sensing

Several single-mode sensing devices exist to detect extravasation. Each one measures a particular physical or physiological factor, including ultrasonic imaging, radiofrequency transmission, ultrasound, fluid pressure, skin strain, and bioimpedance changes (58).

11.1 Impedance Sensing

This system identifies changes in skin impedance. Electrodes or patches are positioned around the catheter insertion site. A small current flows between them, and impedance is measured. A baseline impedance is established before infusion, with subsequent changes tracked. Intravascular infusion results in minimal impedance shifts, whereas fluid accumulation in tissue due to extravasation causes noticeable changes (58).

11.2 Temperature Sensors

Extravasated fluid may change the temperature of nearby tissues, detectable non-invasively with a thermal camera. For greater diagnostic accuracy, infrared thermography can also be used (58).

11.3 Radiofrequency (RF) Sensors

RF signals detect variations in electrical conductivity within soft tissue. Transmitted RF waves pass through the tissue and are received on the other side. Extravasation alters these signals. This method remains effective regardless of tissue temperature or extravasation depth, providing high sensitivity and the ability to identify the leaked fluid (58).

11.4 Optical Sensors

This approach uses light transmitted through the catheter site, with reflected or scattered light detected. Fluid accumulation from extravasation changes the optical density, allowing detection (58).

11.5 Ultrasound Sensors

Ultrasound imaging is essential for confirming catheter tip placement before and during infusion, aiding in the prevention and early detection of extravasation (58).

11.6 Fluid Pressure and Resistance

Fluid resistance and pressure correspond closely with the infusion’s volume and flow rate. Unusual pressure changes during administration may signal complications, such as extravasation (58).

11.7 Pressure–Skin Deformation Sensors

These sensors identify swelling or bulging from fluid leakage into soft tissue. They use multiple skin-contact electrodes. As swelling increases, the distance between electrodes widens, leading to measurable impedance changes (58).

- Multimodal Sensing

Multisensor systems integrate various detection methods to improve diagnostic accuracy. By combining different signals, these systems increase both sensitivity and specificity for early detection of extravasation (58).

12. Differential Diagnosis

Certain intravenous drugs can cause local irritation or venous wall reactions, resulting in symptoms like pain, discomfort, or temporary skin blanching due to venous spasm (30). Not all symptoms at the injection site indicate extravasation (59). Distinguishing extravasation from drug-induced venous reactions is essential:

12.1 Venous Flare Reaction

This short-lived (1–2 hours) superficial venous inflammation appears as streaky, urticaria-like erythema after allergenic drug administration. It is often accompanied by itching and burning but typically lacks severe pain or impaired blood return (23, 61, 62).

12.2 Vessel Irritation

Infusion of irritating agents causes pain, discomfort, skin discoloration, and vein firmness, sometimes with erythema. Unlike extravasation, blood return remains intact (23, 63). Erythema is rare in flare and irritation reactions, whereas extravasation often involves erythema and soft tissue swelling extending beyond the injection site. Limited or absent blood return during infusion is another hallmark of extravasation (63).

12.3 Venous Shock

Rapid infusion or administration of very cold solutions can trigger venous spasm and muscle contraction. Unlike extravasation, there is no skin discoloration or edema. However, blood flow may be reduced, and blood return may be absent. Warm compresses effectively relieve muscle spasms (23).

12.4 Phlebitis

This localized reaction stems from chemical irritation, drug pharmacology, catheter tip infection, or improper catheter size/placement. Symptoms include pain at the infusion site, tenderness, swelling, and vein induration (64).

12.5 Cutaneous Hypersensitivity

This rare immune-mediated reaction typically does not involve tissue toxicity. It presents with mild redness and localized pain but does not progress to severe symptoms (59).

13. Extravasation Kit

Every healthcare facility administering intravenous drugs must keep resources readily available for quick management of potential injuries.*[Table 11]* An extravasation management kit is a critical component, stored in a designated, easily accessible area within each unit. The kit's contents should be routinely inspected for completeness and to ensure no items are expired. Any used or expired items must be promptly replaced through the pharmacy.

The kit includes:

- A treatment protocol and clinical guidelines to enable swift intervention
- Antidotes, medications, and supplies for symptom relief and patient stabilization (1, 14)

Appendix A table 11. Essential Components of Extravasation Emergency Response Kits

**Kit Content**		
**Equipment**	• Chlorhexidine alcohol swabs for site disinfection (14)<br>• Cotton swabs (1)<br>• Sterile gauze and dressings (1)<br>• Permanent black marker (14)	• 3 ml, 5 ml, and 10 ml syringes for drug aspiration and administration (14)<br>• 19- or 21-gauge needle for aspiration from the injection site (1, 14)<br>• 25-gauge needle for injection of antidote (14)<br>• Nitrile gloves (for handling hazardous drugs) (14)
**Symptom Control**	• Five 10 ml ampoules of sterile water for injection (1)<br>• Instant cold and warm compress packs (cold packs labeled "For extravasation only – keep refrigerated") (14)	• One 1500 IU vial of hyaluronidase (1)<br>• One 15 g tube of 1% hydrocortisone cream (1)<br>• Five 10 ml ampoules of 0.9% sodium chloride for injection (1)<br>• Chlorpheniramine: 4 mg oral tablet or 10 mg/ml IV ampoule (14)
**Information and Documentation**	• A drug classification list identifying agents with potential for tissue injury (1)	• A printed, up-to-date copy of the extravasation emergency protocol or Stepwise management algorithm (14)<br>• Extravasation incident report form (1)

14. Initial Therapeutic Management

For mild extravasation injuries, conservative management is advised. In contrast, moderate to severe cases may require surgical intervention (11).

Conservative treatment involves elevating the limb, applying cold or warm compresses, and administering antibiotics, analgesics, and antipyretics. In some instances, airway management, such as tracheostomy, may be necessary (65).

Nurses, being in direct contact with patients, typically begin the initial response. If extravasation is suspected or observed, the infusion should be stopped immediately. Before removing the cannula, attempt to aspirate any residual drug from the site. Supportive measures, antidote administration, and non-compressive dressings, when appropriate, can reduce symptoms and limit tissue damage (17, 66, 67). [Figure 2]

The certainty of evidence supporting these recommendations was assessed qualitatively: Systematic reviews were considered to provide high to moderate certainty evidence (grade B), narrative reviews and expert consensus sources were regarded as moderate certainty evidence (grade C), whereas the observational audit and individual case reports were regarded as low-certainty evidence (grade D).

14.1 Nursing First-Response Checklist

If extravasation is diagnosed

↓

- Confirm extravasation and remove dressing (D)
- Immediately stop infusion and leave the needle in place (C)
- Aspirate the drug using a syringe (C)
- If appropriate and available, administer an antidote (D)
- Remove the cannula (C)
- Elevate the affected limb and apply warm or cold compress as indicated; administer analgesics if necessary (C)
- Marking the affected area or photographing the injury (C)
- Assess severity and notify the attending physician; consult plastic surgery if needed (C)
- Apply appropriate dressing to the affected site, if indicated (C)
- Document the incident (D)
- Monitor and follow up with the patient (C)

Appendix A figure 2. Standardized management protocol for extravasation events

15. Pharmacologic Management

Pharmacologic treatments for extravasation injuries function through two primary mechanisms:

1. **Dilution and dispersion** of the extravasated drug
2. **Neutralization** of the drug with antidotes (28).

15.1 Hyaluronidase

Hyaluronidase is commonly supplied in concentrations of 150 to 200 units/mL. To create a working solution, combine 0.1 mL of the 150-unit/mL formulation with 0.9 mL of 0.9% sodium chloride to achieve a final concentration of 15 units/mL.

Administer 0.2 mL subcutaneously at five sites surrounding the affected area. Additional doses may be given every 30–60 minutes, up to a maximum total dose of 450 units *[Figure 3]*

Note: Intravenous (IV) administration is prohibited (25). To promote absorption and drug dispersion, gently massage the area and apply warm compresses (23).

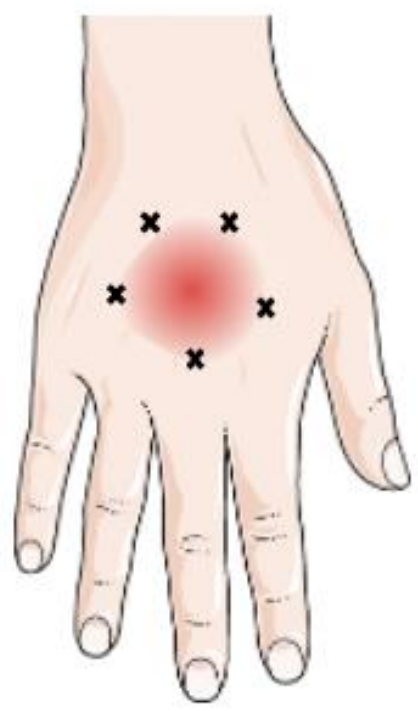

Appendix A figure 3 Injection Sites for Hyaluronidase Administration**,** *Image provided by Servier Medical Art (https://smart.servier.com/), licensed under CC BY 4.0 (https://creativecommons.org/licenses/by/4.0/).*

Mechanism: Hyaluronidase breaks down hyaluronic acid in connective tissue, enhancing tissue permeability and aiding drug diffusion (28). Safety considerations: Hyaluronidase should be administered only by subcutaneous or intradermal injection; intravenous administration is contraindicated. Early administration after recognition of extravasation is recommended to maximize tissue protection (68). Further evaluation is recommended if tissue injury progresses or fails to improve despite hyaluronidase treatment.

Hyaluronidase should be avoided for extravasation of vasoconstrictor agents (e.g., dopamine, norepinephrine, epinephrine, phenylephrine), as it may further expand the area of vasoconstriction rather than mitigate injury. Following administration, the extravasation site should be monitored for swelling, spreading erythema, or signs of hypersensitivity (29, 68).

15.2 Sodium Thiosulfate

To prepare, combine 4 mL of 10% sodium thiosulfate with 6 mL of sterile water for injection or 0.9% sodium chloride to yield a 1.6 molar solution (43). Administer subcutaneously around the injury site, using 2 mL of solution per mg of extravasated drug (17). In calcium-related extravasation, sodium thiosulfate binds to calcium, increasing the solubility of the calcium compound and promoting its removal (69). Safety considerations: Patients receiving sodium thiosulfate should be monitored for non-anion gap metabolic acidosis, hypocalcemia, and severe nausea. Persistent tissue injury or progressive calcinosis warrants specialist evaluation (29).

15.3 Dimethyl Sulfoxide (DMSO)

DMSO encourages vasodilation, stabilizes cell membranes, neutralizes free radicals, and reduces inflammation and pain, helping to prevent tissue necrosis (70). Apply 1–2 mL of a 50–100% solution topically every 4–8 hours for 7–14 days, depending on injury severity. Let the solution air-dry without covering it with occlusive dressings (43)Cold compresses may improve its effectiveness. Avoid using DMSO with dexrazoxane, as it may decrease dexrazoxane's effectiveness (43). Side effects include mild burning, rash, or erythema. Do not apply to intact skin, and wear gloves during application (23, 70). Escalation criteria: Reassessment is recommended if local tissue injury progresses or fails to improve despite treatment (29).

15.4 Dexrazoxane

Dexrazoxane is mainly used for anthracycline-induced extravasation and is not recommended for non-cytotoxic agent extravasations (1).

15.5 Phentolamine

Prepare by diluting 5 mg of phentolamine in 10 mL of normal saline. Inject subcutaneously in 4–5 small doses around the injury site, preferably within 12 hours of extravasation. Repeat every 30–60 minutes if needed (25). As an alpha-

adrenergic antagonist, phentolamine counteracts vasoconstriction caused by extravasated vasopressors, reducing the risk of local ischemia (28). Warm compresses may enhance its effect (28). If phentolamine is unavailable, consider topical nitroglycerin or subcutaneous terbutaline as alternatives (29, 71, 72). Safety considerations: Warm compresses and limb elevation may be used as adjunctive measures, whereas ice packs should be avoided because they may worsen vasoconstriction. If ischemia persists despite treatment, repeat phentolamine administration or alternative vasodilator therapy should be considered (29).

15.6 Nitroglycerin

Nitroglycerin promotes vasodilation by relaxing vascular smooth muscle in arteries and veins (23, 28). Apply a 1-inch topical strip for adults or 4 mm/kg for neonates, reapplying every 8 hours until symptoms resolve (73, 74). Safety considerations: Patients should be monitored for systemic hypotension, particularly neonates and infants receiving repeated topical applications (29). Specialist evaluation is recommended if local ischemia persists or tissue injury progresses despite topical nitroglycerin therapy.

15.7 Terbutaline (β_2-agonist)

Dilute 1 mg of terbutaline in 10 mL of normal saline and inject subcutaneously at five points around the affected site (75). Escalation criteria: If local ischemia does not improve after terbutaline administration, rescue treatment with phentolamine or specialist evaluation should be considered (29). Patients should be monitored for clinical response following subcutaneous administration.

15.8 Antidotes with Uncertain Efficacy

Topical corticosteroids, such as hydrocortisone and dexamethasone, have been used in some cases, but evidence supporting their routine use is limited (30). Additionally, corticosteroids may delay wound healing and increase ulceration risk at high doses, so they are generally not recommended for extravasation injury management (70).

A summary of the preparation and administration of antidotes is provided in [*Table 12].*

Appendix A table 12. Antidote Preparation and Administration Guidelines for Extravasation Management

Antidote	Preparation	Administration
Hyaluronidase	0.1 mL of 150 U/mL + 0.9 mL of 0.9% saline = 15 U/mL (25).	Administer 0.2 mL injections subcutaneously at five sites around the lesion, repeating every 30–60 minutes as needed (25).
Sodium thiosulfate	4 mL of 10% + 6 mL of water or 0.9% saline = 1.6 M solution (43).	Inject 2 mL of the prepared solution per 1 mg of extravasated drug subcutaneously, targeting the area around the extravasation site (17, 43). For calcinosis cutis treatment, administer an intravenous dose of 12.5 grams over 30 minutes, which may be gradually increased to 25 grams three times daily **(29, 76)**.
Dimethyl sulfoxide (DMSO)	-	Apply 1–2 mL topically every 4–8 hours, allowing the solution to air-dry without covering (43). Continue treatment for 7–14 days based on injury severity. Cold compresses are recommended to improve effectiveness (43).
dexrazoxane	-	This antidote is primarily indicated for extravasation injuries caused by anthracyclines (1)
Phentolamine	(5–10) mg Phentolamine +(10–20) mL 0.9% saline (25).	Inject subcutaneously at 4–5 small points around the injury site, ideally within 12 hours of extravasation. If no improvement occurs, repeat the dose every 30–60 minutes (25).
Nitroglycerin topical	1-inch strip = ~7.5 mg (17).	For adults, apply a 1-inch topical strip once daily. For neonates, use 4 mm/kg. Reapply every 8 hours at the injury site if needed until symptoms resolve **(73, 74)**.
Terbutaline	1 mg Terbutaline + 10 mL 0.9% saline (75).	Administer subcutaneously at five injection points around the lesion **(75)**.
Corticosteroids	Not recommended due to risk of delayed healing and ulceration	-

16. Non-Pharmacological Management

One main non-drug method calls for elevating the affected limb for 48 hours to limit or avoid more tissue damage (28). Another common approach includes placing dry warm or cold packs every 4–6 hours for 20 minutes during the first 24–48 hours (23)

In Table 13, the “primary” thermal compress denotes the main, first-choice compress type recommended for a given drug based on its mechanism of tissue injury, while the “secondary” compress denotes an alternative or adjunct option that may also be used, for example when the primary option is unsuitable or as a complementary measure.

16.1 Warm Compress

Warm packs widen local blood vessels, help dilute the drug, and improve spread and uptake of the leaked agent (29, 71, 72). When a vesicant leads to narrowed vessels, warm packs pair with drugs that widen them, such as phentolamine, terbutaline, or nitroglycerin (29, 77)

In addition, warm packs help with leaks of fat-based vesicants (e.g., total parenteral nutrition, propofol) by aiding drug uptake and spread in tissues (25)

16.2 Cold Compress

Cold packs aim to keep the drug in place and block its movement to nearby areas (29, 71, 72). They work well for leaks of blister-causing and cell-killing drugs (78), and they also ease pain and swelling in those instances (28).

The decision between warm or cold packs depends on the drug's way of harming tissues. Importantly, neither heat nor cold goes straight on the skin; always add a layer of dry gauze as a shield (1).

16.3 Manual Massage or Pressure

Manual massage or an air splint can help spread fluid and lower pressure at the site (25).

That said, this method varies by case and sometimes increases harm. In particular, manual pressure must not be used for vesicant drug leaks.

16.4 Surgical Intervention

Mild cases often need only basic care. But moderate to severe ones — especially with ongoing ulcers after basic treatment may require surgery (1, 11).

Surgery brings risks, so proceed carefully and with sound judgment, above all when dead tissue shows and early wash-out fails (11).

✓ 16.4.1 Saline flush-out / Irrigation

This method, like liposuction, uses small cuts, catheter insertion, and suction with saline wash (4).

The patient receives local or general anesthesia. For local, inject 5–10 mL of 1% lidocaine under the skin in and near the site (1).

After that, make five to six upright cuts with a 25G needle into the under-skin layer. Use a cannula to add and remove 500 mL of saline, with 20–30 mL per cut drained from nearby spots (1).*[Figure 4]*

Watch the area every hour for 24 hours. Wrap it in povidone-iodine-soaked gauze, bandage it, and raise it. Add local warmth to aid healing (1).

Best done within 6 hours of harm, though it can help up to 24 hours after. Beyond that, results drop sharply (1).

Keep strict clean methods during the whole process. Repeat saline flush-out should be considered if clinical findings do not improve after the initial procedure. Surgical evaluation is recommended when tissue necrosis is suspected or when inflammatory findings continue to worsen despite treatment (79).

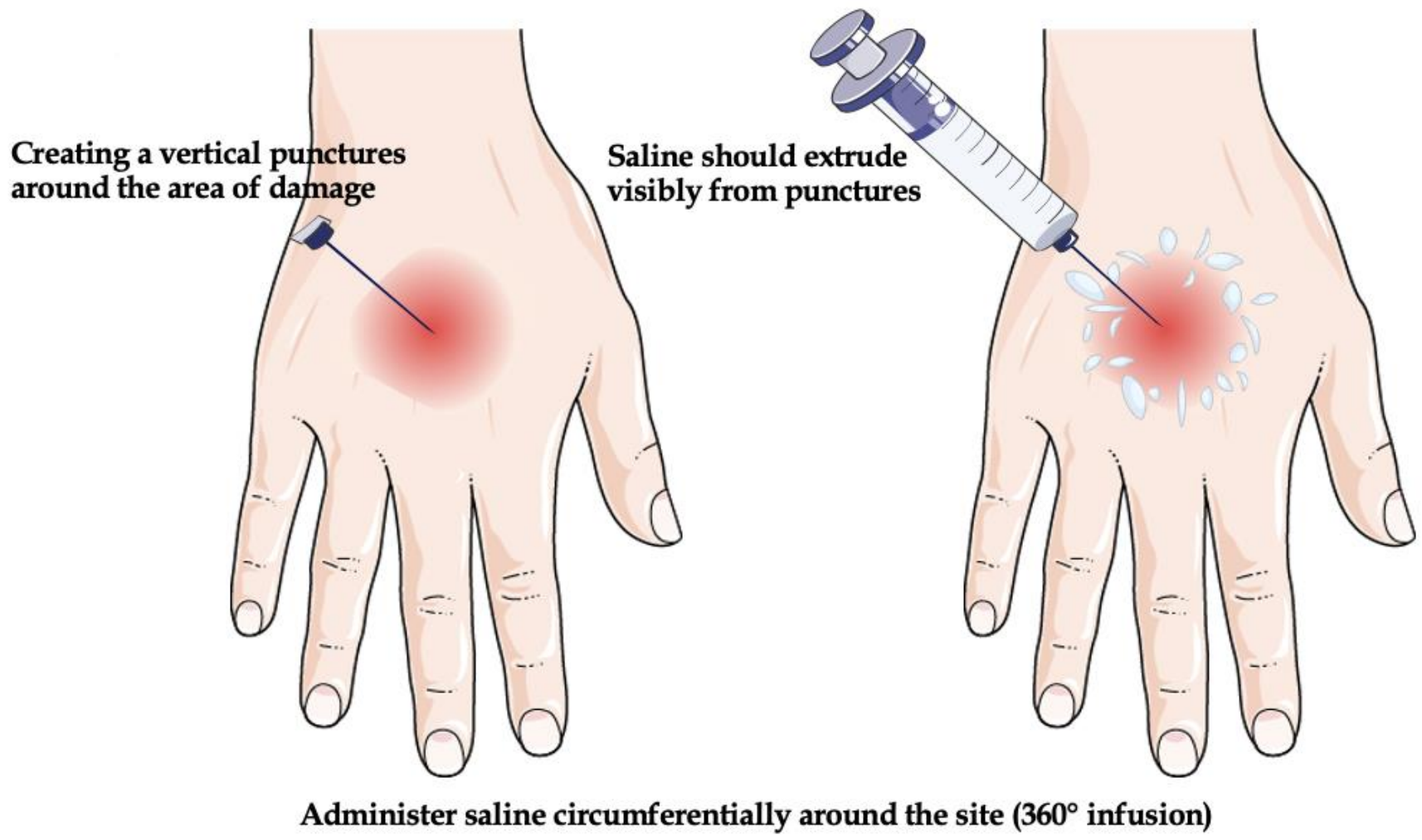

Appendix A figure 4. Normal Saline Flush-Out Irrigation Procedure and Technique**,** *Image provided by Servier Medical Art (https://smart.servier.com/), licensed under CC BY 4.0 (https://creativecommons.org/licenses/by/4.0/).*

✓ 16.4.2 Liposuction

This method requires placing a cannula through side cuts around the injury site to remove subcutaneous fluid and fat (80)

✓ 16.4.3 Debridement and Skin Grafting

If tissue death continues and healing does not occur despite basic care or irrigation methods, surgical removal of dead tissue followed by skin grafting may be necessary.

17. Recommended Management Strategies for Each Drug

Conservative treatment protocols for extravasated drugs, organized by drug classification, are summarized in [Table 13].
Systematic reviews were considered to provide high to moderate certainty evidence (grade B), narrative reviews and expert consensus sources were regarded as moderate certainty evidence (grade C), whereas individual case reports were considered low certainty evidence (grade D).

The strength of each recommendation was determined based on the number and credibility of supporting sources: classified as strong when corroborated by more than three studies, moderate when supported by two reputable studies, and weak when derived from a single source of evidence. Importantly, all recommendations were also experientially validated by the expert panel and practicing nurses. Escalation of care should be guided by clinical severity. In cases of progressive pain, swelling, vascular compromise, or lack of response to initial conservative measures, treatment should be escalated from compress therapy to pharmacologic antidotes. In the presence of signs of tissue ischemia or necrosis, urgent vascular or surgical consultation is indicated.

Appendix A table 13. Conservative Treatment Protocols by Drug Classification

Drug	Thermal compress Primary	Type of evidence And Certainty of evidence	Strength of recommendation	Thermal compress secondary	Type of evidence And Certainty of evidence	Strength of recommendation	Anti dote	Type of evidence And Certainty of evidence	Strength of recommendation
Acetazolamide	-	-	-	-	-	-	-	-	-
Acyclovir	Warm compress (25)	Systematic review citing 1 study (B)	Low	Cold compress (25)	Systematic review citing 1 study (B)	Low	Hyaluronidase (25)	Systematic review citing 2 studies (B)	Moderate
Alprostadil	-	-	-	-	-	-	-	-	-
Alteplase	-	-	-	-	-	-	-	-	-
Amikacin	-	-	-	-	-	-	-	-	-
Aminophylline	Warm compress (25)	Systematic review citing 2 studies (B)	Moderate	Cold or warm compress (25)	Systematic review citing 2 studies (B)	Moderate	Hyaluronidase (25)	Systematic review citing 7 studies (B)	Strong
Amiodarone	Warm compress (25)	Systematic review citing 1 study (B)	Low	Cold or warm compress (25)	Systematic review citing 1 study (B)	Low	Hyaluronidase (25)	Systematic review citing 2 studies (B)	Moderate
Amphotericin B	Cold compress (81)	1 case report (D)	Low	Cold or warm compress (25)	1case report (D)	Low	Hyaluronidase (25)	1 Systematic review (B)	Low

Ampicillin	Warm compress (25)	Systematic review citing 1 study (B)	Low	Cold or warm compress (25)	Systematic review citing 1 study (B)	Low	Hyaluronidase (25)	Systematic review citing 4 studies (B)	Strong
Any medication containing propylene glycol	-	-	-	-	-	-	-	-	-
Arginine	Warm compress(25)	Systematic review citing 1 study (B)	Low	-	-	-	Hyaluronidase (25)	1 Systematic review (B)	Low
Benzylpenicillin	-	-	-	-	-	-	-	-	-
Caffeine citrate	-	-	-	-	-	-	-	-	-
Calcium Gluconate	Warm compress (25)	Systematic review citing 2 studies (B)	Moderate	Cold or warm compress (25)	Systematic review citing 1 study (B)	Low	Early treatment: Hyaluronidase Alternative: sodium thiosulfate (25)	Hyaluronidase: Systematic review citing 8 studies (B) Thiosulfate: Systematic review citing 2 studies (B)	Strong
Calcium chloride>10%	Warm compress (25)	Systematic review citing 2 studies (B)	Moderate	Cold or warm compress (25)	Systematic review citing 1 study (B)	Low	Early treatment: Hyaluronidase Alternative: sodium thiosulfate (25)	Hyaluronidase: Systematic review citing 10 studies (B) Thiosulfate: Systematic review citing 2 studies (B)	Strong
Calcium disodium edetate (EDTA)	Warm compress (25)	Systematic review citing 1 study (B)	Low	-	-	-	Early treatment: Hyaluronidase Alternative: sodium thiosulfate (25)	Hyaluronidase: Systematic review citing 10 studies (B) Thiosulfate: Systematic review citing 2 studies (B)	Strong
Cefazolin	-	-	-	-	-	-	-	-	-
Ceftazidime	-	-	-	-	-	-	-	-	-
Ceftriaxone	-	-	-	-	-	-	-	-	-
Ciprofloxacin	-	-	-	-	-	-	-	-	-
Clindamycin	-	-	-	-	-	-	-	-	-

Conivaptan	Warm compress (25)	Systematic review citing 1 study (B)	Low	-	-	-	Hyaluronidase (25)	Systematic review citing 1 study (B)	Low
Conivaptan	-	-	-	-	-	-	-	-	-
Cytotoxic medications	-	-	-	-	-	-	-	-	-
Dantrolene	Warm compress (25)	Systematic review citing 1 study (B)	Low	-	-	-	Hyaluronidase (25)	Systematic review citing 1 study (B)	Low
Ganciclovir	-	-	-	-	-	-	Topical hydrocortisone (82)	1case report (D)	Low
Glucose > 10%–50%	Warm compress (17)	Systematic review citing 1 study (B)	Low	Cold or warm compress (25)	Systematic review citing 1 study (B)	Low	Hyaluronidase (17)	1 Systematic review (B)	Low
Diazepam	Warm compress (25)	Systematic review citing 1 study (B)	Low	Cold or warm compress(25)	Systematic review citing 1 study (B)	Low	Hyaluronidase (25)	Systematic review citing 1 study (B)	Low
Digoxin	Warm compress (25)	Systematic review citing 1 study (B)	Low	Cold or warm compress(25)	Systematic review citing 1 study (B)	Low	Hyaluronidase (25)	Systematic review citing 1 study (B)	Low
Dobutamine	Warm compress (25)	Systematic review citing 5 studies (B)	Strong	-	-	-	First-line: phentolamine Second-line: terbutaline/topical nitroglycerin (25)	Phentolamine: Systematic review citing 9 studies (B) Terbutaline/topical nitroglycerin: Systematic review citing 3 studies (B)	Strong
Dopamine	Warm compress (25)	Systematic review citing 5 studies (B)	Strong	-	-	-	First-line : phentolamine Second-line : terbutaline/topical nitroglycerin (25)	Phentolamine: Systematic review citing 10 studies (B) Terbutaline: Systematic review citing 3 studies (B) topical nitroglycerin: Systematic review citing 5 studies (B)	Strong

Doxycycline	Warm compress (25)	Systematic review citing 1 study (B)	Low	Cold compress (25)	Systematic review citing 1 studies (B)	Low	Hyaluronidase (25)	Systematic review citing 1 study (B)	Low
Epinephrine	Warm compress (25)	Systematic review citing 5 studies (B)	Strong	-	-	-	First-line : phentolamine Second-line : terbutaline/topical nitroglycerin (25)	Phentolamine: Systematic review citing 11 studies (B) Terbutaline: Systematic review citing 2 studies (B) topical nitroglycerin: Systematic review citing 3 studies (B)	Strong
Epoprostenol	-	-	-	-	-	-	-	-	-
Erythromycin	-	-	-	-	-	-	-	-	-
Esmolol	Warm compress (25)	Systematic review citing 1 study (B)	Low	-	-	-	Hyaluronidase (25)	Systematic review citing 1 study (B)	Low
Etomidate	Warm compress (25)	Systematic review citing 1 study (B)	Low	Cold or warm compress (25)	Systematic review citing 1 study (B)	Low	Hyaluronidase (25)	Systematic review citing 2 studies (B)	Moderate
Fentanyl	-	-	-	-	-	-	-	-	-
Flucloxacillin	-	-	-	-	-	-	-	-	-
Foscarnet	-	-	-	-	-	-	-	-	-
Fosphenytoin	-	-	-	-	-	-	-	-	-
Furosemide	-	-	-	-	-	-	-	-	-
Ganciclovir	-	-	-	-	-	-	-	-	-
Gentamicin	Warm compress (25)	Systematic review citing 4 studies (B)	Strong	-	-	-	Hyaluronidase (25)	Systematic review citing 4 studies (B)	Strong
Heparin	-	-	-	-	-	-	-	-	-
Imipenem/Cilastatin	-	-	-	-	-	-	-	-	-
Immune globulin	Warm compress (25)	Systematic review citing 1 study (B)	Low	-	-	-	Hyaluronidase (25)	Systematic review citing 1 study (B)	Low

Iron infusion	-	-	-	-	-	-	-	-	-
IVIG	-	-	-	-	-	-	-	-	-
Lactated ringers	-	-	-	-	-	-	-	-	-
Lipids	Warm compress (25)	Systematic review citing 1 study (B)	Low	-	-	-	Hyaluronidase (25)	Systematic review citing 1 study (B)	Low
Lorazepam	Warm compress (25)	Systematic review citing 1 study (B)	Low	Cold or warm compress (25)	Systematic review citing 1 study (B)	Low	Hyaluronidase (25)	Systematic review citing 1 study (B)	Low
Magnesium	-	-	-	-	-	-	-	-	-
Mannitol>20%	Warm compress (25)	Systematic review citing 1 study (B)	Low	Cold or warm compress (25)	Systematic review citing 1 study (B)	Low	Hyaluronidase (25)	Systematic review citing 5 studies (B)	Strong
Meropenem	-	-	-	-	-	-	-	-	-
Methylene blue	Warm compress (25)	Systematic review citing 2 studies (B)	Moderate	-	-	-	First-line :topical nitroglycerin Second-line : phentolamine or terbutaline (17)	Topical nitroglycerin: Systematic review citing 2 studies (B) phentolamine or terbutaline: Systematic review citing 2 studies (B)	Moderate
Methylprednisolone	-	-	-	-	-	-	-	-	-
Metronidazole	Warm compress (25)	Systematic review citing 2 studies (B)	Moderate	-	-	-	Hyaluronidase (25)	Systematic review citing 1 study (B)	Low
Midazolam	-	-	-	-	-	-	-	-	-
Morphine	-	-	-	-	-	-	-	-	-
Mycophenolate	-	-	-	-	-	-	-	-	-
Nafcilllin	Warm compress (25)	Systematic review citing 1 study (B)	Low	Cold or warm compress(25)	Systematic review citing 1 study (B)	Low	Hyaluronidase (25)	Systematic review citing 1 study (B)	Low
Nitroglycerin	Cold or warm compress (17)	Systematic review citing 1 study (C)	Low	-	-	-	Hyaluronidase (17)	Systematic review citing 1 study (C)	Low

Norepinephrine	Warm compress (25)	Systematic review citing 5 studies (B)	Strong	-	-	-	First-line : phentolamine Second-line : terbutaline/topica l nitroglycerin (17)	Phentolamine: Systematic review citing 1 study (B) Terbutalin or nitroglycerin: Systematic review citing 3 studies (B)	Strong
Oxacillin	-	-	-	-	-	-	-	-	-
Ondansetron	-	-	-	-	-	-	-	-	-
Penicillin	Warm compress(25)	Systematic review citing 1 study (B)	Low	Cold compress(25)	Systematic review citing 1 study (B)	Low	Hyaluronidase (25)	Systematic review citing 6 studies (B)	Strong
Pentamidine	Warm compress (25)	Systematic review citing 1 study (B)	Low	-	-	-	Hyaluronidase (25)	Systematic review citing 1 study (B)	Low
Pentobarbital	Warm compress (25)	Systematic review citing 1 study (B)	Low	-	-	-	Hyaluronidase (25)	Systematic review citing 1 study (B)	Low
Phenobarbital	Warm compress (25)	Systematic review citing 1 study (B)	Low	Cold compress(25)	Systematic review citing 1 study (B)	Low	Hyaluronidase (25)	Systematic review citing 2 studies (B)	Moderate
Phenylephrine	Warm compress (25)	Systematic review citing 3 studies (B)	Strong	-	-	-	First-line: phentolamine Second-line: topical nitroglycerin(17)	Phentolamine: Systematic review citing 10 studies (B) Topical nitroglycerin: Systematic review citing 3 studies (B)	Strong
Phenytoin and fosphenytoin	Warm compress (17)	Systematic review citing 2 studies (B)	Moderate	-	-	-	Hyaluronidase or nitroglycerin(17)	Systematic review citing 2 studies (B)	Moderate
Phosphate salts	Warm compress (25)	Systematic review citing 1 study (B)	Low	Cold or warm compress(25)	Systematic review citing 1 study (B)	Low	-	-	-
Piperacillin/Tazo bactam	-	-	-	-	-	-	-	-	-

Potassium chloride	Warm compress (25)	Systematic review citing 2 studies (B)	Moderate	Cold or warm compress(25)	Systematic review citing 1 studies (B)	Low	Hyaluronidase (25)	Systematic review citing 9 studies (B)	Strong
Potassium phosphate	Cold compress (17)	Systematic review citing 1 study (B)	Low	-	-	-	Hyaluronidase (17)	Systematic review citing 1 study (B)	Low
Promethazine	Warm compress (25)	Systematic review citing 1 study (B)	Low	Cold compress(25)	Systematic review citing 1 studies (B)	Low	Hyaluronidase (25)	Systematic review citing 1 study (B)	Low
Propofol	Warm compress (25)	Systematic review citing 1 study (B)	Low	Cold compress(25)	Systematic review citing 1 studies (B)	Low	Hyaluronidase (25)	1 Systematic review (B)	Low
Sodium bicarbonate	Warm compress (25)	Systematic review citing 3 studies (B)	Moderate	Cold or warm compress(25)	Systematic review citing 1 studies (B)	Low	Hyaluronidase (25)	Systematic review citing 6 studies (B)	Strong
Sodium chloride>3%	Warm compress (25)	Systematic review citing 1 study (B)	Low	Cold or warm compress(25)	Systematic review citing 1 studies (B)	Low	Hyaluronidase (25)	Systematic review citing 5 studies (B)	Strong
Sodium Thiopental	-	-	-	-	-	-	-	-	-
Sodium phosphate	Warm compress (17)	Systematic review citing 1 study (B)	Low	-	-	-	Hyaluronidase (25)	Systematic review citing 1 study (B)	Low
Sulfate	-	-	-	-	-	-	-	-	-
Tobramycin	-	-	-	-	-	-	-	-	-
Total parental nutrition (TPN)	Warm compress (25)	Systematic review citing 1 study (B)	Low	Cold or warm compress(25)	Systematic review citing 2 studies (B)	Moderate	Hyaluronidase , nitroglycerin(17)	Systematic review citing 10 studies (B)	Strong
Valproate	Warm compress (25)	Systematic review citing 1 study (B)	Low	-	-	-	Hyaluronidase (25)	1 Systematic review (B)	Low
Vancomycin	Warm compress (25)	Systematic review citing 2 studies (B)	Moderate	Cold compress(25)	Systematic review citing 1 studies (B)	Low	Hyaluronidase (25)	Systematic review citing 9 studies (B)	Strong
Vasopressin	Warm compress (25)	Systematic review citing 2 studies (B)	Moderate	-	-	-	First-line: topical nitroglycerin Second-line: phentolamine or terbutaline(17)	Topical nitroglycerin: Systematic review citing 1 study (B)	Strong

								Terbutalin or Phentolamine: Systematic review citing 3 studies (B)	

Legend. Recommendation strength reflects the relative amount of supporting literature. Strength of recommendation: Strong = supported by more than 3 sources; Moderate = supported by 2 sources; Weak = supported by 1 source. Certainty of evidence: Grade B = systematic review (high-to-moderate certainty); Grade C = clinical guideline, expert consensus, or narrative review (moderate-to-low certainty); Grade D = individual case report (low certainty). "-" indicates insufficient evidence to support a specific recommendation for that drug. Primary: preferred first line thermal compress; Secondary: alternative or adjunct option, when clinically appropriate.

18. Follow-Up

Patients with suspected extravasation require evaluation within 1 to 3 days of symptom onset to assess the severity and scope of tissue damage. Final treatment choices hinge on new signs or worsening of current symptoms. Hospitalized patients must promptly report any worsening of lesions to ensure timely treatment for confirmed or delayed extravasation (1).

After hourly checks in the first 24 hours, patients with confirmed extravasation need daily assessments for the initial days, followed by weekly checks to track recovery. Key signs to watch include pain, ulceration, blisters, and redness. Depending on injury severity and the drug involved, monitoring may extend from 1–2 to 4–7 weeks until full recovery. For discharged patients, followup visits are necessary to check lesion progress (1, 83).

To prevent delays in possible surgery, look for warning signs like increasing redness, ulceration, widespread blisters, or ongoing severe pain (1, 31). Apply sterile dressings to blistered or necrotic areas to avoid secondary infections, and prescribe suitable pain relief for those with intense pain (1). Even patients with mild injuries managed conservatively need ongoing monitoring and prompt symptom reporting (30).

Finally, provide clear education to patients and caregivers, both verbally and in writing (83). For those under conservative care, give precise guidance on using warm or cold packs, elevating the limb, and scheduling follow-ups. Patients must know the signs requiring referral—such as swelling, redness, blistering, skin breakdown, or pain—and whom to contact. For those with a central venous catheter (CVC), additional symptoms like fever, chest pain, or cough also warrant referral (46).

19. Documentation and Patient Records

Recording an extravasation event is a vital task that must be done quickly after the initial emergency response, while ensuring urgent treatments are not delayed. *[Table 14]*

Appendix A table 14. Mandatory Documentation Requirements for Extravasation Incidents

Record Components	
Number of IV insertion attempts and failed cannulations(32)	Patient identification data (32)
Concurrent medications administered during the same timeframe (6)	Date and time of extravasation occurrence (32)
Management and treatment strategies applied (32)	Type, concentration, and dose of the extravasated drug **(83 ,32)**
Follow-up plan and ongoing care (32)	Device and method of administration (e.g., peripheral or central catheter, pump infusion), including details such as type, length, and gauge of catheter (32)
Photographic documentation of the lesion site (32)	Exact site of drug administration (32)
Checklist of written and verbal patient education, including warning signs that require medical attention (83)	Estimated volume of extravasated drug (6)
Signature of the attending nurse and/or physician (32)	Patient-reported symptoms and clinical signs observed by healthcare staff **(32, 83)**

20. Investigational Treatments

20.1 Vacuum-Assisted Closure (VAC)

VAC therapy applies negative pressure to remove leaked fluid from tissues. Data remain limited, but some studies report good results when added to other care (15).

20.2 Hyperbaric Oxygen Therapy (HBO)

The way HBO acts in extravasation injuries is not fully known. It seems to aid healing by raising reactive oxygen species. Evidence is sparse, yet a few studies indicate partial success (15).

20.3 Amniotic Membrane

Amniotic membrane acts as a fresh biological dressing for extravasation wounds and lessens the call for skin grafts (84). It holds anti-inflammatory and anti-angiogenic proteins, along with precursors such as endostatin and thrombospondin-1. Place the membrane over the wound and check it every 48 hours. Replace it every five days, with treatment averaging 2.5 weeks based on wound size (84).

No adverse effects or infections appear in reports. Since wound care stays simple, it pleases doctors and nurses. The method suits neonates well, as early signs are hard to spot and many adult options do not apply (84).

The inner placental layer provides strong biological and immune features that speed recovery. It counters inflammation, bacteria, fibrosis, and pain. Amniotic epithelial and mesenchymal cells skip immune reactions, which keeps them safe (84).

Mainly applied to diabetic ulcers and burns, amniotic membrane could help deep wounds as well, though studies stay scarce (85).

Commercial options range from dehydrated and cryopreserved to amnion-only and amnion/chorion types (86).

Examples:

- Amicor: A dehydrated amniotic membrane for chronic wounds.
- Ecurge_C: A cryopreserved liquid membrane used in burns and ophthalmic surgery.
- Equirage_C: A lyophilized, room-temperature stable liquid amniotic product.
- Acellular: A wet amniotic liquid designed for burn-related wounds (85).

20.4 Active Leptospermum Honey (ALH)

ALH handles necrotic, infected wounds that produce exudate (87)

In open wounds, ALH works best as a gel or hydrocolloid. Its acid level reduces bacterial presence and raises oxygen flow. High osmolarity from ALH boosts lymph drainage, aids self-debridement, curbs bacterial growth, and cuts inflammatory mediators and proteases. Plus, it delivers antimicrobial action through hydrogen peroxide (88)

Honey-based topical items, such as gels and non-stick dressings, support wound recovery, but stubborn wounds might demand more involved care (89).

21. Reference

1. White S, Robinson J, Barr E. Chemotherapy extravasation guideline–WOSCAN (West of Scotland Cancer Advisory Network) Cancer Nursing and Pharmacy Group), Date written: September 2009.
2. Gong Z, Zhang J, Hou J, Chen S, Hu Z, Kong X, et al. Drug Extravasation in a Large General Hospital in Hunan, China: A Retrospective Survey. Risk Manag Healthc Policy. 2021;14:4931-8.
3. Lynch DJ, Key JC, White RRt. Management and prevention of infiltration and extravasation injury. Surg Clin North Am. 1979;59(5):939-49.
4. Gault DT. Extravasation injuries. Br J Plast Surg. 1993;46(2):91-6.
5. Kumar RJ, Pegg SP, Kimble RM. Management of extravasation injuries. ANZ J Surg. 2001;71(5):285-9.
6. Hadaway L. Infiltration and extravasation. Am J Nurs. 2007;107(8):64-72.
7. Lamoreux MR, Sternbach MR, Hsu WT. Erythema multiforme. Am Fam Physician. 2006;74(11):1883-8.
8. Farthing P, Bagan JV, Scully C. Mucosal disease series. Number IV. Erythema multiforme. Oral Dis. 2005;11(5):261-7.
9. Higginson R, Parry A. Phlebitis: treatment, care and prevention. Nurs Times. 2011;107(36):18-21.
10. Lv L, Zhang J. The incidence and risk of infusion phlebitis with peripheral intravenous catheters: A meta-analysis. J Vasc Access. 2020;21(3):342-9.
11. Alexander L. Extravasation Injuries: A Trivial Injury Often Overlooked with Disastrous Consequences. World J Plast Surg. 2020;9(3):326-30.
12. Hedayatipour A, Aslanzadeh S, McFarlane N. CMOS based whole cell impedance sensing: Challenges and future outlook. Biosens Bioelectron. 2019;143:111600.
13. Melbourne TRCsH, Year. Peripheral extravasation injuries: Initial management and washout procedure Melbourne: The Royal Children's Hospital; 2023 [Available from: https://www.rch.org.au/clinicalguide/guideline_index/Peripheral_extravasation_injuries__Initial_management_and_washout_procedure/.
14. Executive HS. NCCP Guidance: Prevention and Management of Extravasation of Systemic Anti-Cancer Therapy (SACT). Dublin: National Cancer Control Programme; 2021 [Version 1:[Available from: https://www.hse.ie/eng/services/list/5/cancer/profinfo/medonc/sactguidance/nccp-extravasation-guidance.pdf
.
15. Kreidieh FY, Moukadem HA, El Saghir NS. Overview, prevention and management of chemotherapy extravasation. World J Clin Oncol. 2016;7(1):87-97.
16. Ersan SA, Şen A. Overview of the Pathophysiology of Extravasation of Anesthetic Drugs. Journal of the Society of Thoracic Carido-Vascular Anaesthesia & Intensive Care. 2024;30(1).
17. Tarpey A, Narechania S, Malesker MJCCjom. What is the optimal approach to infiltration and extravasation of nonchemotherapy medications? 2023;90(5):292-6.
18. Shibata Y, Taogoshi T, Matsuo H. Extravasation of Noncytotoxic Agents: Skin Injury and Risk Classification. Biol Pharm Bull. 2023;46(6):746-55.
19. Scales K. Arterial catheters: indications, insertion and use in critical care. Br J Nurs. 2010;19(19):S16-21.
20. Chopra V, Flanders SA, Saint S, Woller SC, O'Grady NP, Safdar N, et al. The Michigan Appropriateness Guide for Intravenous Catheters (MAGIC): Results From a Multispecialty Panel Using the RAND/UCLA Appropriateness Method. Ann Intern Med. 2015;163(6 Suppl):S1-40.
21. Onesti MG, Carella S, Fioramonti P, Scuderi N. Chemotherapy Extravasation Management: 21-Year Experience. Ann Plast Surg. 2017;79(5):450-7.
22. Maly C, Fan KL, Rogers GF, Mitchell B, Amling J, Johnson K, et al. A primer on the acute management of intravenous extravasation injuries for the plastic surgeon. 2018;6(4):e1743.
23. Kim JT, Park JY, Lee HJ, Cheon YJJJoeefhp. Guidelines for the management of extravasation. 2020;17.

24. Dufficy M, Takashima M, Cunninghame J, Griffin BR, McBride CA, August D, et al. Extravasation injury management for neonates and children: A systematic review and aggregated case series. 2022;17(10):832-42.
25. Ong J, Van Gerpen RJJoIN. Recommendations for management of noncytotoxic vesicant extravasations. 2020;43(6):319-43.
26. Billingham MJ, Mittal R. Peripheral venous extravasation injury. BJA Educ. 2023;23(2):42-5.
27. Chen TK, Yang CY, Chen SJ. Calcinosis cutis complicated by compartment syndrome following extravasation of calcium gluconate in a neonate: a case report. Pediatr Neonatol. 2010;51(4):238-41.
28. Smolders EJ, Benoist GE, Smit CCH, Ter Horst P. An update on extravasation: basic knowledge for clinical pharmacists. European Journal of Hospital Pharmacy. 2021;28(3):165-7.
29. Reynolds PM, MacLaren R, Mueller SW, Fish DN, Kiser TH. Management of extravasation injuries: a focused evaluation of noncytotoxic medications. Pharmacotherapy. 2014;34(6):617-32.
30. Lake C, Beecroft CLJCEiA, Critical Care, Pain. Extravasation injuries and accidental intra-arterial injection. 2010;10(4):109-13.
31. Gonzalez TJCJoON. Chemotherapy extravasations: prevention, identification, management, and documentation. 2013;17(1).
32. Pikó B, Laczó I, Szatmári K, Bassam AA, Szabó Z, Ócsai HH, et al. Overview of extravasation management and possibilities for risk reduction based on literature data. 2013;3(9):93.
33. Antúnez-Blancat A, Gago-Valiente F-J, García-Iglesias J-J, Merino-Navarro D, editors. The Role of Nursing in the Management of Chemotherapy Extravasation: A Systematic Review Regarding Public Health. Healthcare; 2024: MDPI.
34. Barber CM, Fahrenkopf MP, Adams NS, Kelpin JP, Krebiehl JR. Compartment Syndrome of the Hand Secondary to Intravenous Extravasation. Eplasty. 2018;18:ic17.
35. Elliott KG, Johnstone AJ. Diagnosing acute compartment syndrome. J Bone Joint Surg Br. 2003;85(5):625-32.
36. Hargens AR, Botte MJ, Swenson MR, Gelberman RH, Rhoades CE, Akeson WH, et al. Effects of local compression on peroneal nerve function in humans. J Orthop Res. 1993;11(6):818-27.
37. Whitesides TE, Heckman MM. Acute Compartment Syndrome: Update on Diagnosis and Treatment. J Am Acad Orthop Surg. 1996;4(4):209-18.
38. Ko CH, Tay SY, Chang HC, Chan WP. Large-volume iodinated contrast medium extravasation: low frequency and good outcome after conservative management in a single-centre cohort of more than 67,000 patients. Eur Radiol. 2018;28(12):5376-83.
39. van Veelen NM, Link BC, Donner G, Babst R, Beeres FJP. Compartment syndrome of the forearm caused by contrast medium extravasation: A case report and review of the literature. Clin Imaging. 2020;61:58-61.
40. Grand A, Yeager B, Wollstein R. Compartment syndrome presenting as ischemia following extravasation of contrast material. Can J Plast Surg. 2008;16(3):173-4.
41. Clark E, Giambra BK, Hingl J, Doellman D, Tofani B, Johnson NJJoIN. Reducing risk of harm from extravasation: a 3-tiered evidence-based list of pediatric peripheral intravenous infusates. 2013;36(1):37-45.
42. Kassner E. Evaluation and treatment of chemotherapy extravasation injuries. J Pediatr Oncol Nurs. 2000;17(3):135-48.
43. Schulmeister L, editor Extravasation management: clinical update. Seminars in oncology nursing; 2011: Elsevier.
44. Albanell J, Baselga J, editors. Systemic therapy emergencies. Seminars in oncology; 2000.
45. Bertelli G. Prevention and management of extravasation of cytotoxic drugs. Drug Saf. 1995;12(4):245-55.
46. Sauerland C, Engelking C, Wickham R, Corbi D, editors. Vesicant extravasation part I: Mechanisms, pathogenesis, and nursing care to reduce risk. Oncology nursing forum; 2006.
47. Boschi R, Rostagno E. Extravasation of antineoplastic agents: prevention and treatments. Pediatr Rep. 2012;4(3):e28.
48. Anderson NR. When to use a midline catheter. Nursing. 2005;35(4):28.
49. Anderson NR. Midline catheters: the middle ground of intravenous therapy administration. J Infus Nurs. 2004;27(5):313-21.
50. Society IN. Infusion Nursing Standards of Practice (2011): Untreed Reads; 2011.

51. Inker LA, Astor BC, Fox CH, Isakova T, Lash JP, Peralta CA, et al. KDOQI US commentary on the 2012 KDIGO clinical practice guideline for the evaluation and management of CKD. Am J Kidney Dis. 2014;63(5):713-35.
52. Levey AS, Coresh J, Bolton K, Culleton B, Harvey KS, Ikizler TA, et al. K/DOQI clinical practice guidelines for chronic kidney disease: evaluation, classification, and stratification. American Journal of Kidney Diseases. 2002;39(2 SUPPL. 1):i-ii+ S1-S266.
53. Kellum JA, Lameire N, Group KAGW. Diagnosis, evaluation, and management of acute kidney injury: a KDIGO summary (Part 1). Crit Care. 2013;17(1):204.
54. Coresh J, Turin TC, Matsushita K, Sang Y, Ballew SH, Appel LJ, et al. Decline in estimated glomerular filtration rate and subsequent risk of end-stage renal disease and mortality. JAMA. 2014;311(24):2518-31.
55. El Ters M, Schears GJ, Taler SJ, Williams AW, Albright RC, Jenson BM, et al. Association between prior peripherally inserted central catheters and lack of functioning arteriovenous fistulas: a case-control study in hemodialysis patients. Am J Kidney Dis. 2012;60(4):601-8.
56. Sasadeusz KJ, Trerotola SO, Shah H, Namyslowski J, Johnson MS, Moresco KP, et al. Tunneled jugular small-bore central catheters as an alternative to peripherally inserted central catheters for intermediate-term venous access in patients with hemodialysis and chronic renal insufficiency. Radiology. 1999;213(1):303-6.
57. Deutsch GB, Sathyanarayana SA, Singh N, Nicastro J. Ultrasound-guided placement of midline catheters in the surgical intensive care unit: a cost-effective proposal for timely central line removal. J Surg Res. 2014;191(1):1-5.
58. Hirata I, Mazzotta A, Makvandi P, Cesini I, Brioschi C, Ferraris A, et al. Sensing Technologies for Extravasation Detection: A Review. ACS Sens. 2023;8(3):1017-32.
59. De Wit M, Ortner P, Lipp H-P, Sehouli J, Untch M, Ruhnke M, et al. Management of cytotoxic extravasation-ASORS expert opinion for diagnosis, prevention and treatment. Oncology Research and Treatment. 2013;36(3):127-35.
60. Mayer A, Zholkover A, Keidan I, editors. A functional test for the detection of infusion lines extravasation. 2014 36th Annual International Conference of the IEEE Engineering in Medicine and Biology Society; 2014: IEEE.
61. Gill JM, Frost J, Park R, Saria MG. Venous Flare Reactions: A case report of reactions following etoposide infusion. Clinical journal of oncology nursing. 2018;22(6).
62. Vogelzang N. " Adriamycin flare": a skin reaction resembling extravasation. Cancer Treatment Reports. 1979;63(11-12):2067-9.
63. Wengstrom Y, Margulies A, European Oncology Nursing Society Task F. European Oncology Nursing Society extravasation guidelines. Eur J Oncol Nurs. 2008;12(4):357-61.
64. Webster J, McGrail M, Marsh N, Wallis MC, Ray-Barruel G, Rickard CM. Postinfusion Phlebitis: Incidence and Risk Factors. Nurs Res Pract. 2015;2015(1):691934.
65. Quintanar Verduguez T, Blanco Jarava A, Martinez-Barbeito MB, Pangua Mendez C, Lopez Gomez L, Andrade Santiago J, et al. Mediastinal extravasation of doxorubicin. Clin Transl Oncol. 2008;10(2):128-30.
66. Goutos I, Cogswell LK, Giele H. Extravasation injuries: a review. J Hand Surg Eur Vol. 2014;39(8):808-18.
67. Aziz A, Samoon Z, Shaheen Z, Feroz A, Khurshid M, Ayoub N, et al. Audit on incidents and knowledge of nurses regarding chemotherapy extravasations at day care oncology of a tertiary care hospital in Karachi. Pakistan Journal of Oncology and Cancer Research. 2019;3(1).
68. Hanrahan K. Hyaluronidase for treatment of intravenous extravasations: implementation of an evidence-based guideline in a pediatric population. Journal for Specialists in Pediatric Nursing. 2013;18(3):253-62.
69. Compaña FJP, Míguez JM, de Toro Santos FJ, Cortés AC, San Martín PL, Vidal MTY-P, et al. The use of antidotes for calcium gluconate extravasation: an experimental study in mice. Plastic and Reconstructive Surgery. 2018;142(3):699-707.
70. Wickham R, Engelking C, Sauerland C, Corbi D, editors. Vesicant extravasation part II: Evidence-based management and continuing controversies. Oncology Nursing Forum; 2006.
71. David V, Christou N, Etienne P, Almeida M, Roux A, Taibi A, et al. Extravasation of Noncytotoxic Drugs. Ann Pharmacother. 2020;54(8):804-14.
72. Le A, Patel S. Extravasation of Noncytotoxic Drugs: A Review of the Literature. Ann Pharmacother. 2014;48(7):870-86.

73. Wong AF, McCulloch LM, Sola A. Treatment of peripheral tissue ischemia with topical nitroglycerin ointment in neonates. J Pediatr. 1992;121(6):980-3.
74. Denkler KA, Cohen BE. Reversal of dopamine extravasation injury with topical nitroglycerin ointment. Plast Reconstr Surg. 1989;84(5):811-3.
75. Stier PA, Bogner MP, Webster K, Leikin JB, Burda A. Use of subcutaneous terbutaline to reverse peripheral ischemia. Am J Emerg Med. 1999;17(1):91-4.
76. Raffaella C, Annapaola C, Tullio I, Angelo R, Giuseppe L, Simone C. Successful treatment of severe iatrogenic calcinosis cutis with intravenous sodium thiosulfate in a child affected by T-acute lymphoblastic leukemia. Pediatr Dermatol. 2009;26(3):311-5.
77. Martin SM. Extravasation management of nonchemotherapeutic medications. J Infus Nurs. 2013;36(6):392-6.
78. Santivasi WL, Kulkarni S, Patton ML, Haith LR, Guilday RE, Reigart CL, et al. Infiltration of sodium valproate with compartment syndrome and bullous reaction: case report and literature review. Burns. 2011;37(7):e59-62.
79. Steiert A, Hille U, Burke W, Gohritz A, Zilz S, Herold C, et al. Subcutaneous wash-out procedure (SWOP) for the treatment of chemotherapeutic extravasations. Journal of plastic, reconstructive & aesthetic surgery. 2011;64(2):240-7.
80. Corbett M, Marshall D, Harden M, Oddie S, Phillips R, McGuire W. Treatment of extravasation injuries in infants and young children: a scoping review and survey. Health Technol Assess. 2018;22(46):1-112.
81. Olson C, McCoy LK. Amphotericin B extravasation: a case report. Journal of Infusion Nursing. 1985;8(4):299-300.
82. Powell CW, Borrelli C, White W, Carson T. Ganciclovir-Induced Bullae Formation Secondary to Extravasation in a Patient Being Treated for Cytomegalovirus Retinitis. Ann Pharmacother. 2018;52(12):1265-6.
83. Nicola R, Shaqdan KW, Aran S, Prabhakar AM, Singh AK, Abujudeh HH. Contrast Media Extravasation of Computed Tomography and Magnetic Resonance Imaging: Management Guidelines for the Radiologist. Curr Probl Diagn Radiol. 2016;45(3):161-4.
84. Kadivar M. Evaluation efficacy of amniotic membrane in the treatment of neonatal Extravasation: a pilot study. 2021.
85. Babak A, Ansari K. Aminotic Membrane Dressing for Chemotherapy Extravasation Wounds. Journal of Clinical Case Studies Reviews & Reports SRC/JCCSR-218 J Clin Stud Rev Rep. 2023;5(6):2-.
86. Boyar V, Galiczewski C. Efficacy of Dehydrated Human Amniotic Membrane Allograft for the Treatment of Severe Extravasation Injuries in Preterm Neonates. Wounds. 2018;30(8):224-8.
87. Raisi Z, Fard SB, Fard SB, Khabazzadeh F. Management of Extravasation Injury in Infants: A Systematic Review of Clinical Trials. Medical-Surgical Nursing Journal. 2023;12(1).
88. Molan P, Rhodes T. Honey: A Biologic Wound Dressing. Wounds. 2015;27(6):141-51.
89. Mayer D, Armstrong D, Schultz G, Percival S, Malone M, Romanelli M, et al. Cell salvage in acute and chronic wounds: a potential treatment strategy. Experimental data and early clinical results. J Wound Care. 2018;27(9):594-605.

Supplementary file 1 (Search Strategies)

Designed Search Queries for Scientific Databases and Search Engines.

Database/Search Engine	Syntax	Number of Records Found
PubMed (Medline)	("Extravasation of Diagnostic and Therapeutic Materials"[Mesh] OR extravasation*[tiab] OR "Tissue infiltration"[tiab] OR "intravenous infiltration"[tiab] OR "injection site reaction*"[tiab] OR paravasation*[tiab]) AND (prevent*[tiab] OR manag*[tiab] OR treatment*[tiab] OR "clinical practice guideline*"[tiab] OR protocol*[tiab] OR antidote*[tiab] OR "catheter selection"[tiab])	2,902
Scopus	TITLE-ABS-KEY (extravasation* OR "tissue infiltration" OR "intravenous infiltration" OR "Injection site reaction*" OR paravasation*) AND TITLE-ABS-KEY (prevent* OR manag* OR treatment* OR "clinical practice guideline*" OR protocol* OR antidote* OR "catheter selection")	2,964
Web of Science (Clarivate)	TS= (extravasation* OR "tissue infiltration" OR "intravenous infiltration" OR "Injection site reaction*" OR paravasation*) AND TS= (prevent* OR manag* OR treatment* OR "clinical practice guideline*" OR protocol* OR antidote* OR "catheter selection")	1,724

Supplementary File 2 (Delphi Questionnaire)

- **Delphi Questionnaire (Round 1)**

Form No. 1 – Topic Identification

Objective: To assess the necessity of each proposed topic for inclusion in the clinical guideline on non-chemotherapeutic drug extravasation.

No.	Proposed Topic	Is it necessary?	Suggestion or Revision
1	Introduction	☐ Yes ☐ No	…………………
2	Definitions	☐ Yes ☐ No	…………………
3	Symptoms at different severity levels	☐ Yes ☐ No	…………………
4	Mechanism of injury	☐ Yes ☐ No	…………………
5	Consequences of extravasation	☐ Yes ☐ No	…………………
6	Risk factors	☐ Yes ☐ No	…………………
7	Prevention methods	☐ Yes ☐ No	…………………
8	Diagnosis	☐ Yes ☐ No	…………………
9	Differential diagnosis	☐ Yes ☐ No	…………………
10	Extravasation kit	☐ Yes ☐ No	…………………
11	Immediate initial actions	☐ Yes ☐ No	…………………
12	Pharmacological treatment	☐ Yes ☐ No	…………………
13	Non-pharmacological treatment	☐ Yes ☐ No	…………………
14	Recommended interventions for each drug	☐ Yes ☐ No	…………………
15	Documentation	☐ Yes ☐ No	…………………
16	Follow-up	☐ Yes ☐ No	…………………
17	Your proposed topic(s):	…………………………………………………………	

- **Delphi Questionnaire (Round 2)**

Form No. 2 – Feedback on the Initial Draft of the Guideline

Objective: To evaluate the structure, scientific accuracy, and clinical applicability of the guideline developed by the expert team.

Section Reviewed	Evaluation Question	Acceptable?	Suggestions or Revisions
Overall structure	Is the text organized and systematically structured?	☐ Yes ☐ No	…………………
Language and writing	Is the text linguistically fluent and technically accurate?	☐ Yes ☐ No	…………………
Content coverage	Does the guideline comprehensively address all necessary aspects?	☐ Yes ☐ No	…………………
Clinical applicability	Is this guideline suitable for practical use in hospitals?	☐ Yes ☐ No	…………………
Chapter arrangement	Are the order and titles of the topics appropriate and logical?	☐ Yes ☐ No	…………………
Appropriateness of treatments	Are the pharmacological and non-pharmacological treatments applicable and effective?	☐ Yes ☐ No	…………………
Additional suggestions	Are there any points that should be added or removed?	☐ Yes ☐ No	…………………

No.	Proposed Topic	Is it necessary?	Suggestion or Revision
1	Introduction	☐ Yes ☐ No	…………………
2	Definitions	☐ Yes ☐ No	…………………
3	Symptoms at different severity levels	☐ Yes ☐ No	…………………
4	Mechanism of injury	☐ Yes ☐ No	…………………
5	Consequences of extravasation	☐ Yes ☐ No	…………………
6	Risk factors	☐ Yes ☐ No	…………………
7	Prevention methods	☐ Yes ☐ No	…………………
8	Diagnosis	☐ Yes ☐ No	…………………
9	Differential diagnosis	☐ Yes ☐ No	…………………

10	Extravasation kit	☐ Yes ☐ No	…………………
11	Immediate initial actions	☐ Yes ☐ No	…………………
12	Pharmacological treatment	☐ Yes ☐ No	…………………
13	Non-pharmacological treatment	☐ Yes ☐ No	…………………
14	Recommended interventions for each drug	☐ Yes ☐ No	…………………
15	Documentation	☐ Yes ☐ No	…………………
16	Follow-up	☐ Yes ☐ No	…………………
17	Detector	☐ Yes ☐ No	…………………
18	Catheter selection	☐ Yes ☐ No	…………………
19	Proposed treatment strategies	☐ Yes ☐ No	…………………
20	A risk-based classification table	☐ Yes ☐ No	…………………
21	Your proposed topic(s):	…………………………………………………………	

- **Delphi Questionnaire (Round 3)**

Form No. 3 – Final Version Approval

Objective: To confirm the clinical guideline after revisions based on Round 2 feedback.

Aspect	Final approval granted?	If not, please explain
Structure, organization, and writing of the guideline	☐ Yes ☐ No	…………………
Scientific accuracy of the content	☐ Yes ☐ No	…………………
Clinical and practical applicability	☐ Yes ☐ No	…………………
Overall satisfaction with the final version	☐ Yes ☐ No	…………………

No.	Proposed Topic	Is it necessary?	Suggestion or Revision
1	Introduction	☐ Yes ☐ No	…………………
2	Definitions	☐ Yes ☐ No	…………………
3	Symptoms at different severity levels	☐ Yes ☐ No	…………………
4	Mechanism of injury	☐ Yes ☐ No	…………………
5	Consequences of extravasation	☐ Yes ☐ No	…………………
6	Risk factors	☐ Yes ☐ No	…………………
7	Prevention methods	☐ Yes ☐ No	…………………
8	Diagnosis	☐ Yes ☐ No	…………………
9	Differential diagnosis	☐ Yes ☐ No	…………………
10	Extravasation kit	☐ Yes ☐ No	…………………
11	Immediate initial actions	☐ Yes ☐ No	…………………
12	Pharmacological treatment	☐ Yes ☐ No	…………………
13	Non-pharmacological treatment	☐ Yes ☐ No	…………………

14	Recommended interventions for each drug	☐ Yes ☐ No	…………………
15	Documentation	☐ Yes ☐ No	…………………
16	Follow-up	☐ Yes ☐ No	…………………
17	Detector	☐ Yes ☐ No	…………………
18	Catheter selection	☐ Yes ☐ No	…………………
19	Proposed treatment strategies	☐ Yes ☐ No	…………………
20	A risk-based classification table	☐ Yes ☐ No	…………………
21	Your proposed topic(s):	………………………………………………………	

Supplementary file 3 (flow chart)

Flow chart illustrating the stages of the Delphi processe

Initial Topic Selection and Draft Preparation

- Identification of necessary topics for inclusion in the guideline based on a review of previous guidelines and existing gaps.
- Data collection from literature and preparation of the preliminary draft of the guideline.
- Selection of the expert panel with relevant clinical and academic experience.

↓

Round 1

- Distribution of the initial guideline draft along with the Round 1 questionnaire to all panel members.
- Collection of feedback and responses from each panel member.
- Review of feedback and incorporation of required revisions into the draft.

↓

Round 2

- Distribution of the revised draft along with the Round 2 questionnaire.
- Collection of panel feedback and responses.
- Review and implementation of necessary modifications.

↓

Round 3

- Distribution of the further revised draft with the Round 3 questionnaire.
- Collection of feedback and responses from panel members.
- Final review and incorporation of modifications to achieve consensus.

↓

Consensus Confirmation

- Final approval from all expert panel members.
- Confirmation that consensus has been reached on all items.

↓

External Validation

- Submission of the guideline to external experts for independent review.
- Collection of external feedback regarding clarity, relevance, applicability, and usability.
- Incorporation of suggested improvements.

↓

Finalization

- Review of all modifications suggested by external reviewers.
- Preparation of the final version of the guideline ready for dissemination.

Supplementary file 4 (External Questionnaire)

External Validation Form — Delphi-Based Clinical Practice Guideline

No.	Section	Evaluation Criteria	Acceptable?	Suggestions or Revisions
1	Clarity	The content and recommendations are clearly presented and easy to understand.	Yes ☐ No☐	
2	Coherence	The recommendations are logically structured and internally consistent.	Yes ☐ No☐	
3	Practical Applicability	The guideline can be feasibly implemented in real clinical settings.	Yes ☐ No☐	
4	Completeness	The guideline covers all necessary domains and interventions.	Yes ☐ No☐	
5	Structure and format	The organization of sections and flow of content are logical and user-friendly.	Yes ☐ No☐	
6	Additional Comments			
